# Efficient carbon-Ferrier rearrangement on glycals mediated by ceric ammonium nitrate: Application to the synthesis of 2-deoxy-2-amino-*C*-glycoside

**DOI:** 10.3762/bjoc.10.27

**Published:** 2014-01-30

**Authors:** Alafia A Ansari, Y Suman Reddy, Yashwant D Vankar

**Affiliations:** 1 Department of Chemistry, Indian Institute of Technology Kanpur 208 016, India

**Keywords:** C-glycosides, carbon-Ferrier rearrangement, ceric ammonium nitrate, 2-deoxy-2-aminoglycosides, Overman rearrangement

## Abstract

A carbon-Ferrier rearrangement on glycals has been performed by using ceric ammonium nitrate to obtain products in moderate to good yields with high selectivity. The versatility of this method has been demonstrated by applying it to differently protected glycals and by employing several nucleophiles. The obtained *C*-allyl glycoside has been utilized for the synthesis of a orthogonally protected 2-amino-2-deoxy-*C*-glycoside.

## Introduction

The growing significance of *C*-glycosides can be attributed to their potential use as inhibitors of carbohydrate-processing enzymes [1–3], their extraordinary stability compared to *O*-glycosides, and their widespread applicability as intermediates in the synthesis of biologically important molecules [4–8]. *C*-glycosides are also subunits of several biologically active natural products [9–11]. Consequently, numerous reports are available in literature on the synthesis of *C*-glycosides [12–14]. Among these, the Ferrier rearrangement [15] of glycals with protic acids [5,16–17] or Lewis acids [18–22] and carbon nucleophiles such as allylsilanes [23], silylacetylenes [24], silyl enol ethers [25], olefins [26], and organozinc reagents [27] has emerged as a popular method. In particular, *C*-allyl glycosides, glycosyl cyanides, and glycosyl azides have received considerable attention, as the allyl, cyanide and azide moieties can be readily converted into a variety of other functional groups [6,28–30]. Furthermore, the resulting *C*-pseudoglycals are also important as the double bond can also be easily functionalized in a variety of ways.

Although there is an abundance of methods reporting on the carbon-Ferrier rearrangement of glycals [9], efforts are ongoing for improvements in terms of efficiency, selectivity, time and yields of reaction. In view of our continued interest in the development of novel methods for the synthesis of *O*- and *C*-glycosides [31–36], we here report on the carbon-Ferrier rearrangement of glycals by using ceric ammonium nitrate. Ceric ammonium nitrate (CAN) is a versatile and efficient reagent, which has been well-explored for a variety of reactions in literature [37–38]. CAN was utilized successfully in carbohydrate chemistry for important transformations such as the azidonitration of glycals and the formation of 2-C-branched glycosides from glycals [39–41]. In this paper, we report on the addition of carbon nucleophiles onto differently protected glycals by using CAN. The corresponding Ferrier rearrangement products were obtained in moderate to fairly good yields and with a high selectivity.

## Results and Discussion

We initially performed the carbon-Ferrier rearrangement on 3,4,6-tri-*O*-acetyl-D-glucal (**1a**) by using 3 equivalents of allyltrimethylsilane and 2 equivalents of CAN in anhydrous acetonitrile at room temperature. The reaction proceeded smoothly over 1 hour and exclusively furnished the α-*C*-allyl glycoside **2a** in 88% yield (Table 1) [42]. We attempted the same reaction with a catalytic amount (10–30 mol %) of CAN. However, under these conditions the reaction showed complicated TLC profiles and did not complete even after overnight stirring or heating under reflux for several hours. Changing the solvent to dichloromethane slowed down the reaction considerably due to the poor solubility of CAN, whereas in acetone the reaction gave lower yields. After several optimization experiments, the reaction was observed to be most efficient when 2 equivalents of silane and one equivalent of CAN, with respect to the glycal, was used in acetonitrile.

**Table 1:** Optimization of reaction conditions.

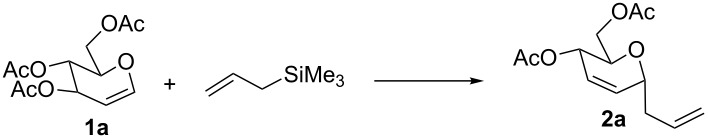						

Entry	Silane (equiv)	CAN (equiv)	Solvent	Temp (°C)	Time (h)	Yield (%)

1	3.0	2.0	CH_3_CN	rt	1 h	88
2	3.0	0.2	CH_3_CN	reflux	12 h	38
3	3.0	0.5	CH_3_CN	reflux	12 h	45
4	3.0	1.0	CH_3_CN	rt	1.5 h	82
5	2.0	1.0	CH_3_CN	rt	2 h	88
6	2.0	1.0	CH_3_COCH_3_	rt	6 h	53
7	2.0	1.0	CH_2_Cl_2_	rt	12 h	20

The reaction was then carried out with several glycals (Table 2). and yielded a single product in all cases studied. In the case of glycals **1a–d**, only the α-anomer was obtained as has been reported by Danishefsky et al. in the first report on the *C*-glycosidation of glycals with allyltrimethylsilane by using TiCl_4_ [23]. The spectral data of the obtained *C*-allyl glycosides supported the known data [42–44]. In general, D-glucals were found to react faster than D-galactals and provided the rearranged products in less time and better yields. Moreover, even the benzylated glycals **1c** and **1d** readily underwent the Ferrier rearrangement under these conditions. The pentose glycals **1e–h** exclusively gave the *anti*-products, confirming previous investigations [45]. Glycals **1i**, with a TBDPS protecting group, and **1k**, with a methyl protecting group, were also found to undergo the reaction in good yields affording **2i** (see Supporting Information File 1) and **2k** [42], respectively. These findings indicate that the protecting groups were not affected by this reagent system. D-rhamnal **1j** and D-ribose derived glycal **1l** [46] yielded hitherto unknown *C*-allyl glycosides **2j** and **2l**, respectively (see Supporting Information File 1). However, the same reaction with benzylidene or isopropylidene glucals resulted in a complex mixture of products and the desired product was not isolated.

**Table 2:** Reaction of glycals with allyltrimethylsilane by using CAN.

Entry	Glycal	Product	Time	Yield	Ref.

1	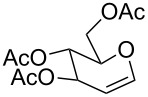 **1a**	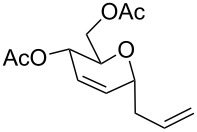 **2a**	2 h	86	[42]
2	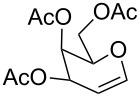 **1b**	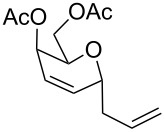 **2b**	3 h	75	[42]
3	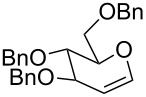 **1c**	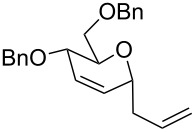 **2c**	4 h	69	[42]
4	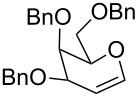 **1d**	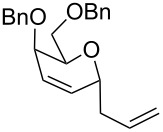 **2d**	8 h	52	[44]
5	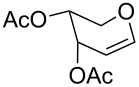 **1e**	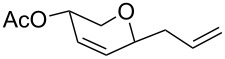 **2e**	2 h	80	[43,45]
6	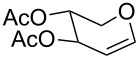 **1f**	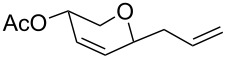 **2f**	3 h	84	[43,45]
7	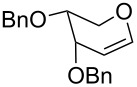 **1g**	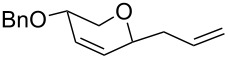 **2g**	4 h	71	[46]
8	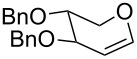 **1h**	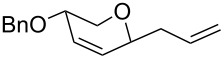 **2g**	8 h	61	[46]
9	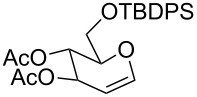 **1i**	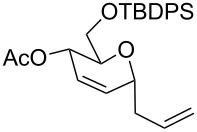 **2i**	4 h	67	[46]
10.	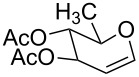 **1j**	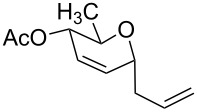 **2j**	4 h	66	[43]
11.	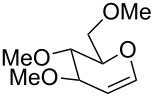 **1k**	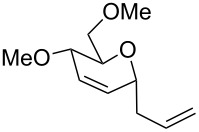 **2k**	2 h	78	[43]
12.	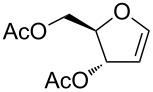 **1l**	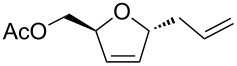 **2l**	3 h	62	[46]

Furthermore, the scope of this reaction was extended by subjecting glycals to reactions with other nucleophiles. Thus, glycals **1a** and **1b** subjected to treatment with Me_3_SiCN afforded a 5:1 and 4:1 mixture of glycosyl cyanides **3** and **4** in 77% and 62% yields, respectively (Table 3). The reaction of glycals **1a** and **1b** with Me_3_SiN_3_ furnished a mixture of C-1 and C-3 substituted glycosyl azides (**5/5'** and **6/6'**). This observation is in conformity with the report of Hayashi and co-workers [47], which described the obtainment of Ferrier products along with C-3 substituted products. On the other hand, glucal **1a** on reaction with triethylsilane afforded the product **7** in 43% yield, while galactal **1b** gave a complex mixture of products, which could not be analyzed.

**Table 3:** Reaction of glycals with other nucleophiles.

Entry	Glycal	Nucleophile	Product(s)	Time	Yield	Ref.

1	**1a**	TMSCN	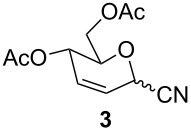	6 h	77	[42]
2	**1b**	TMSCN	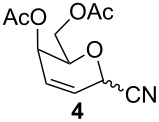	12 h	62	[42]
3	**1a**	TMSN_3_	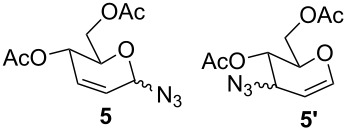	3 h	69	[42,47]
4	**1b**	TMSN_3_	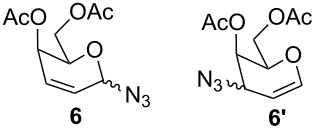	4 h	59	[42,47]
5	**1a**	Et_3_SiH	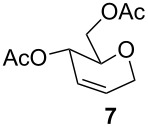	2 h	43	[43]

## Proposed mechanism

**Scheme 1:**
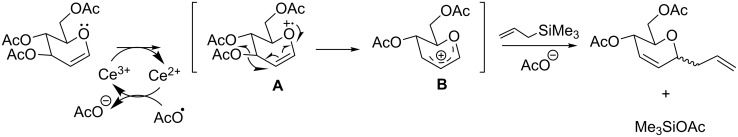
Proposed mechanism.

The proposed mechanism of the formation of the *C*-allyl glycosides is shown in Scheme 1. The ring oxygen could donate an electron to the Ce^3+^ leading to the formation of a radical cation **A**. Subsequent migration of the double bond and loss of the acetyl radical could result in the formation of the delocalized carbocation **B**. The acetate radical could accept an electron from Ce^2+^ thereby forming an acetate anion, which could in turn attack the silyl moiety of allyltrimethylsilane leading to the formation of trimethylsilyl acetate and an allyl anion. The allyl anion could then attack at the anomeric position so that the C-allyl pseudoglycoside is fomed. However, we were not able to isolate Me_3_SiOAc. As the acetate ion and the acetyl radical are better leaving groups the formation of the acyl radical seems to be more facile than the formation of the alkoxy radicals (e.g., benzyloxy and methoxy). This explains faster reaction times in the case of acetylated glycals compared to benzyl or methyl glycals. It is well-known in the literature that the participation of a neighboring group is possible in the case of glucals thereby leading to a higher reactivity than the corresponding galactals [48–49].

Having obtained the α*-C*-glycosides in an efficient manner, we explored their synthetic utility to synthesize a 2-deoxy-2-amino-α-*C*-glycoside. 2-Deoxy-2-amino-α-*C*-glycosides have received considerable attention in recent years due to their use in the synthesis of glycopeptides [50–51], glycolipids [51] and glycosyl amino acids [52] to name but a few. Amongst the various reported methods to prepare these compounds [53–55], the most common method is via C-glycosylation of 2-aminosugars [56–58], which is challenging owing to the incompatibility of protic or Lewis acids with amino and amido functionality. In particular, very few reports on the synthesis of orthogonally protected C-glycosides appeared in literature [59]. Therefore, there is a need for the development of efficient methods to allow easy access to this important class of compounds.

**Scheme 2:**
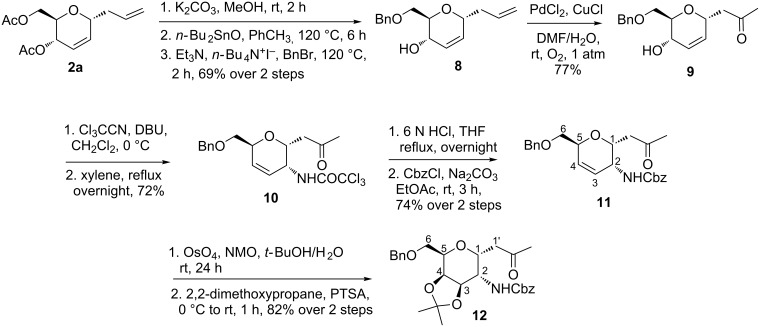
Synthesis of 2-deoxy-2-amino-*C*-glycoside **12** from Ferrier product **2a**.

For this purpose, Ferrier rearranged product **2a** was subjected to deacetylation by using K_2_CO_3_/MeOH (Scheme 2) followed by the selective protection of the primary hydroxy group as a benzyl ether by using Bu_2_SnO and benzyl bromide in the presence of triethylamine and tetrabutylammonium iodide to afford alcohol **8** in 69% yield over 2 steps. The terminal olefin in compound **8** was then subjected to a Wacker oxidation [60] by using a catalytic amount of PdCl_2_ and an excess of CuCl under O_2_ atmosphere to obtain the methyl ketone **9** in a good yield. The formation of the product was confirmed by the disappearance of the external olefinic protons and the appearance of a sharp singlet at δ 2.11, corresponding to methyl protons in the ^1^H NMR spectrum, as well as a peak at δ 207 ppm in the ^13^C NMR spectrum corresponding to the carbonyl group (see Supporting Information File 1). The allylic alcohol **9** was converted to the corresponding trichloroacetimidate, which in crude form was heated under reflux in xylene, to afford the single isomer **10** in 72% yield by an Overman rearrangement [61–63].

The trichloroacetamide group was hydrolyzed by heating under reflux in 6 N HCl, and the obtained free amine was protected as a benzyloxycarbamate group by using benzyl chloroformate and Na_2_CO_3_. The protected amide **11** was obtained in 74% yield over 2 steps. The regioselectivity of the Overman rearrangement step was determined from the ^1^H NMR and COSY experiments of compound **11**. These experiments showed that the anomeric proton (H-1) at δ 4.40 correlated with the H-2 proton at δ 4.00, which in turn was adjacent to the amide proton at δ 4.85, thereby indicating that the amide moiety is present at the C-2 position (see Supporting Information File 1). The internal olefin was subjected to a dihydroxylation under the Upjohn conditions [64], followed by an acetonide protection of the resulting diol by using 2,2-dimethoxypropane and a catalytic amount of *para*-toluenesulfonic acid (PTSA) to furnish the desired orthogonally protected 2-deoxy-2-amino-*C*-glycoside **12** as a single isomer in 82% yield (over 2 steps).

**Figure 1:**
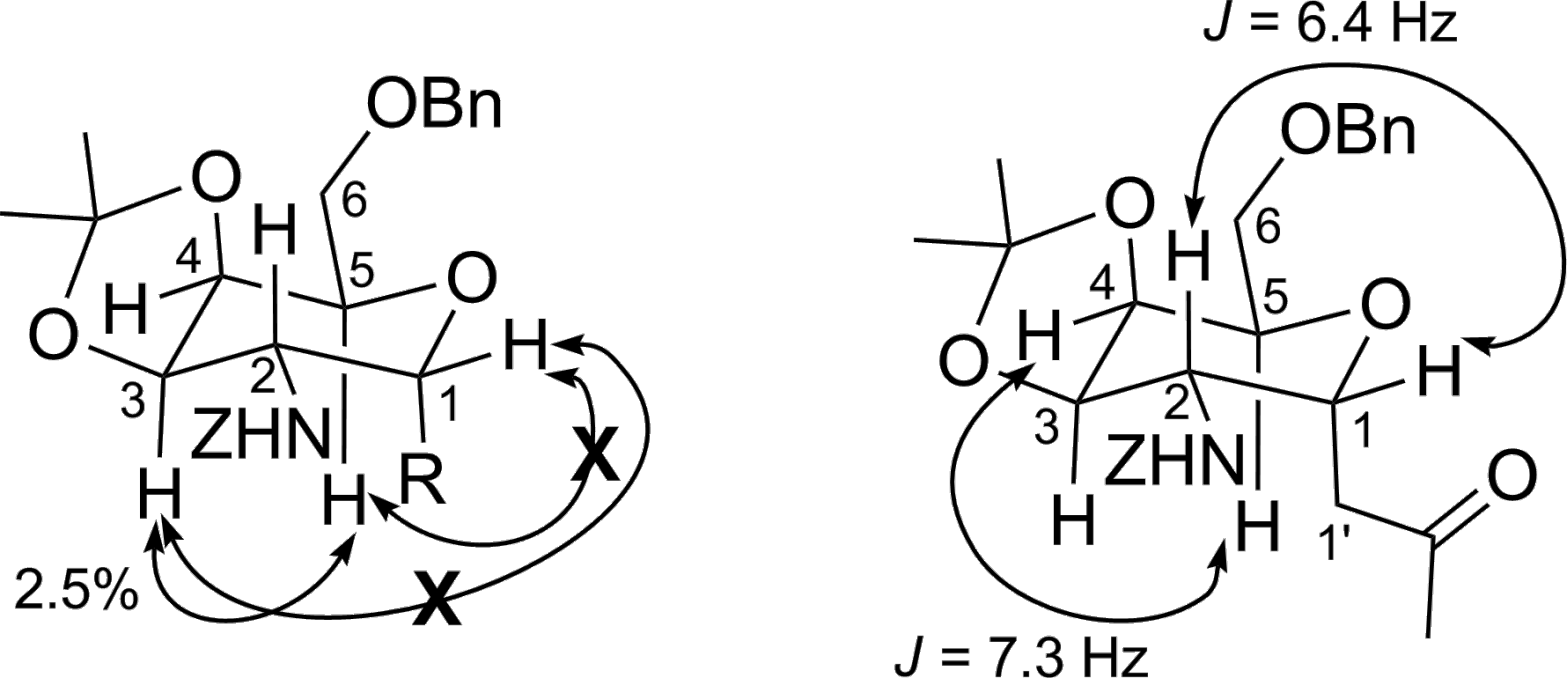
nOe and decoupling experiments of compound **12**.

The stereochemistry of the newly generated stereocenters in compound **12** was determined with the help of ^1^H NMR, COSY, nOe and decoupling experiments (Figure 1). Thus, the irradiation of the signal at δ 3.93 corresponding to H-3 led to an enhancement of the H-5 signal at δ 4.25 by 2.5%, while the peak at δ 4.63, corresponding to H-1, was not enhanced. This implies that the H-3 proton is *cis* to H-5 and *trans* to H-1 and thus axially oriented. Meanwhile, the irradiation of the H-1 signal at δ 4.63 did not give any enhancement of the H-3 and H-5 peaks at δ 3.93 and δ 4.25, respectively. Moreover, the homonuclear decoupling of the H-3 signal at δ 3.93 gave the coupling constant of H-4 and H-5 as *J* = 7.3 Hz, which indicates an axial-equatorial relationship. The decoupling of the H-1' signals at δ 2.55 provided the coupling constant of H-1 and H-2 as *J* = 6.4 Hz, indicating an axial-equatorial relationship (see Supporting Information File 1). This suggests that the rearrangement took place from the axial side, as expected, since the hydroxy group at C-4 is axially oriented in compound **9**. Moreover, the dihydroxylation took place from the side opposite to the amino group at C-2, which can be attributed to the steric hindrance from the bulky amide group.

A D-galacto-configured C-glycoside **12** was obtained from D-glucal **1a**, which may serve as a versatile synthetic intermediate, since the carbonyl moiety and the double bond functionality in this compound can be synthetically manipulated in a variety of ways.

## Conclusion

We have developed an efficient method for the Ferrier rearrangement of glycals by using ceric ammonium nitrate and several carbon nucleophiles. We have successfully employed the obtained *C*-allyl glycoside **2a** for the stereoselective synthesis of a orthogonally protected 2-deoxy-2-amino-*C*-glycoside **12** via an Overman rearrangement as a key step.

## Supporting Information

File 1 Analytical data and copies of the ^1^H NMR and ^13^C NMR spectra of all new compounds.
