# Supplementary material for: Efficient carbon-Ferrier rearrangement on glycals mediated by ceric ammonium nitrate: Application to the synthesis of 2-deoxy-2-amino-C-glycoside
Source: Beilstein J Org Chem. 2014 Jan 30;10:300–6. doi: 10.3762/bjoc.10.27 (PMC3943741; doi:10.3762/bjoc.10.27)
Supplement: File 1 — Analytical data and copies of the 1H NMR and 13C NMR spectra of all new compounds. [file Beilstein_J_Org_Chem-10-300-s001.pdf]

# Supporting Information

# for

# Efficient carbon-Ferrier rearrangement on glycals mediated by ceric ammonium nitrate: Application to the synthesis of 2-deoxy-2-amino-C-glycoside

Alafia A. Ansari, Y. Suman Reddy and Yashwant D. Vankar*

Address: Department of Chemistry, Indian Institute of Technology Kanpur 208 016, India

Email: Yashwant D. Vankar - vankar@iitk.ac.in

*Corresponding author

**Analytical data and copies of the $^{1}$H NMR and $^{13}$C NMR spectra of all new compounds**

**Contents**


| | |
|---|---|
| Experimental section: General methods | S2 |
| Analytical data of the compounds | S3–S10 |
| Copies of NMR spectra | S11–S26 |

## Experimental section

**General methods:** All the experiments have been carried out in oven-dried apparatus and in dry solvents under nitrogen atmosphere unless indicated otherwise. Commercial grade solvents were dried by methods known in literature and stored over 4 Å molecular sieves. TLC plates were prepared by using thin layers of silica gel on microscopic slides and the visualization of spots was effected by exposure to iodine or spraying with 10% $H_2SO_4$ and charring. Column chromatography was performed over silica gel (100–200 mesh) by using hexane and ethyl acetate as an eluent. Optical rotations were measured with a polarimeter at 28 °C in the indicated solvents. IR spectra were recorded as a thin film and expressed in $cm^{-1}$. $^{1}H$ NMR (400 or 500 MHz) and $^{13}C$ NMR (100 or 125 MHz) spectra were recorded with $CDCl_3$ as a solvent. Chemical shifts were reported in ppm downfield to tetramethylsilane and coupling constants expressed in Hertz (Hz). Splitting patterns were assigned as s (singlet), d (doublet), dd (doublet of doublet), dt (doublet of triplet), td (triplet of doublet), m (multiplet) or br (broad). Mass spectra were obtained by Q-TOF by using the electrospray ionisation method.

**General procedure for Ferrier rearrangement of glycals by using CAN:** A glycal (0.368 mmol) was dissolved in freshly dried $CH_3CN$ (3 mL) under $N_2$ atmosphere. To this solution was added a nucleophile (0.736 mmol), followed by ceric ammonium nitrate (202 mg, 0.368 mmol). The reaction mixture was stirred at room temperature for the time indicated. After complete consumption of glycal (TLC monitoring), the reaction mixture was poured into a saturated $NaHCO_3$ solution (5 mL), and extracted with ethyl acetate (3 × 5 mL). Combined organic extracts were washed with brine (1 × 10 mL), dried over $Na_2SO_4$, and concentrated under vacuum. The obtained residue was purified by column chromatography.

**(3*S*,6*S*)-6-Allyl-3-(benzyloxy)-3,6-dihydro-2*H*-pyran (2g):** Glycals **1g** and **1h** were subjected to the reaction following the general procedure described above. **2g** was obtained in 71% and 61% yields, respectively, as a colorless oil: $R_f$ = 0.6 (hexane/EtOAc = 19:1); $[\alpha]_D^{28}$ = +71.4 (*c* 0.70, $CH_2Cl_2$); IR (neat) $\nu_{max}$: 3032, 2921, 2859, 1641, 1496, 1454, 1100 $cm^{-1}$; $^1H$ NMR (500 MHz, $CDCl_3$): $\delta$ 7.35–7.25 (m, 5H), 5.93 (dd, *J* = 3.4, 10.8 Hz, 1H), 5.85–5.77 (m, 2H), 5.12–5.07 (m, 2H), 4.63 (d, *J* = 11.7 Hz, 1H), 4.57 (d, *J* = 11.7 Hz, 1H), 4.16–4.07 (m, 3H), 3.48 (dd, *J* = 7.1, 10.0 Hz, 1H), 2.29–2.25 (m, 2H); $^{13}C$ NMR (125 MHz, $CDCl_3$): $\delta$ 138.4, 134.2, 131.7, 128.5, 127.8, 127.6, 127.0, 117.4, 73.6, 71.1, 69.6, 67.1, 39.4; HRMS calcd for $C_{15}H_{18}NaO_2$ $[M + Na]^+$ 253.1204, Found: 253.1208.

**(2*R*,3*S*,6*R*)-6-Allyl-2-((*tert*-butyldiphenylsilyloxy)methyl)-3,6-dihydro-2*H*-pyran-3-yl acetate (2i)** : Compound **2i** was obtained from glycal **1i** (50 mg, 0.11 mmol) in 67% yield (36 mg) as a colorless oil: $R_f$ = 0.4 (hexane/EtOAc = 19:1); $[\alpha]_D^{28}$ = +36.0 (*c* 1.00, $CH_2Cl_2$); IR (neat) $\nu_{max}$: 3072, 2931, 2858, 1741, 1472, 1428, 1370, 1235, 1112 $cm^{-1}$; $^1H$ NMR (500 MHz, $CDCl_3$): $\delta$ 7.68–7.66 (m, 4H), 7.43–7.36 (m, 6H), 5.90–5.77 (m, 3H), 5.13–5.07 (m, 3H), 4.18 (*br* s, 1H), 3.91 (dd, *J* = 5.1, 10.9 Hz, 1H), 3.79–3.75 (m, 2H), 2.43 (dd, *J* = 6.3, 13.7 Hz, 1H), 2.29 (dd, *J* = 7.7, 13.7 Hz, 1H), 2.00 (s, 3H), 1.05 (s, 9H); $^{13}C$ NMR (125 MHz, $CDCl_3$): $\delta$ 170.5, 135.7, 134.3, 133.4, 129.7, 127.7, 123.3, 117.4, 73.5, 70.7, 65.3, 63.2, 38.4, 26.8, 21.2, 19.3; HRMS calcd for $C_{27}H_{38}NO_4Si$ $[M + NH_4]^+$ 468.2570, Found: 468.2574.

**((2*S*,5*R*)-5-Allyl-2,5-dihydrofuran-2-yl)methyl acetate (2l):** Ribose glycal **1l** (50 mg, 0.25 mmol) was converted to **2l** (28mg, 62%). Colorless oil: $R_f$ = 0.5 (hexane/EtOAc = 9:1); $[\alpha]_D^{28}$ = +110.0 (*c* 0.60, $CH_2Cl_2$); IR (neat) $\nu_{max}$: 2923, 2853, 1732, 1445, 1011 $cm^{-1}$; $^1H$ NMR (500 MHz, $CDCl_3$): $\delta$ 5.90 (dt, *J* = 1.7, 10.6 Hz, 1H), 5.85–5.77 (m, 2H), 5.25–5.21 (m, 1H), 5.13–5.07 (m, 2H), 4.19–4.15 (m, 1H), 4.11 (dd, *J* = 4.9, 11.4 Hz, 1H), 3.53 (dd, *J* = 6.6, 11.4

Hz, 1H), 2.37–2.24 (m, 2H), 2.05 (s, 3H); $^{13}$C NMR (125 MHz, $CDCl_3$): $\delta$ 170.1, 133.9, 133.6, 124.5, 117.7, 73.1, 65.1, 65.0, 38.7, 31.3; HRMS calcd for $C_{10}H_{14}NaO_3$ [M + Na]$^+$ 205.0841 Found: 205.0846.

**Compound 3**: The glycal **1a** (100 mg, 0.37 mmol) was treated with TMSCN (0.09 mL, 0.74 mmol) by using the general procedure to afford **3** (74 mg, 77%) as a colorless oil: $R_f$ = 0.5 (hexane/EtOAc = 3:1); IR (neat) $\nu_{max}$: 3645, 3473, 2923, 2133, 1732, 1434, 1370, 1220, 1092 cm$^{-1}$; $^1$H NMR (5:1 mixture of diastereomers, 500 MHz, $CDCl_3$): $\delta$ 6.35 (m, 1H, minor isomer), 6.27 (dd, $J$ = 2.5, 11.3 Hz, 1H, major isomer), 6.14 (m, 1H, minor isomer), 6.08 (dd, $J$ = 1.9, 11.3 Hz, 1H, major isomer), 5.19 (br s, 2H, major isomer), 5.02 (br s, 2H, minor isomer), 4.23 (m, 3H, major isomer), 3.98 (m, 3H, minor isomer), 2.14-2.07 (m, 3H, both isomers); $^{13}$C NMR (5:1 mixture of diastereomers, 100 MHz, $CDCl_3$): $\delta$ 172.1, 171.9, 170.3, 170.1, 132.0, 129.8, 123.8, 122.4, 116.2, 72.1, 64.3, 62.5, 62.1, 60.2, 58.1, 57.6, 22.5, 22.1, 20.5, 20.4; HRMS calcd for $C_{11}H_{13}NNaO_5$ [M + Na]$^+$ 262.0691, Found: 262.0691.

**Compound 4**: The glycal **1b** (100 mg, 0.37 mmol) was treated with TMSCN (0.09 mL, 0.74 mmol) by using the general procedure to afford **4** (60 mg, 62%) as a colorless oil: $R_f$ = 0.5 (hexane/EtOAc = 3:1); IR (neat) $\nu_{max}$: 3640, 3477, 2925, 2132, 1735, 1438, 1369, 1209, 1105 cm$^{-1}$; $^1$H NMR (4:1 mixture of diastereomers, 500 MHz, $CDCl_3$): $\delta$ 6.33 (m, 1H, minor isomer), 6.24 (dd, $J$ = 1.7, 8.0 Hz, 1H, major isomer), 6.04 (m, 1H, both isomers), 5.13 (br s, 2H, both isomers), 4.25 (m, 3H, major isomer), 4.03 (m, 3H, minor isomer), 2.14-2.07 (m, 3H, both isomers); $^{13}$C NMR (125 MHz, $CDCl_3$): $\delta$ 171.3, 170.7, 170.1, 126.5, 126.4, 124.7, 115.3, 115.2, 72.2, 72.0, 62.7, 62.2, 62.0, 60.8, 21.9, 20.8, 20.7; HRMS calcd for $C_{11}H_{13}NNaO_5$ [M + Na]$^+$ 262.0691, Found: 262.0691.

**Compound 5**: The glycal **1a** (100 mg, 0.37 mmol) was treated with $TMSN_3$ (0.10 mL, 0.74 mmol) by using the general procedure to afford **5** (74 mg, 69%) as a colorless oil: $R_f$ = 0.5 (hexane/EtOAc = 4:1); IR (neat) $\nu_{max}$: 2922, 2851, 2104, 1746, 1646, 1454, 1371, 1223 $cm^{-1}$; $^1$H NMR (500 MHz, $CDCl_3$): $\delta$ 6.52-6.48 (m, 1H), 5.94 (d, J = 10.3 Hz, 1H), 5.79-5.76 (m, 1H), 5.56 ( br s, 1H), 5.32-5.30 (m, 1H), 5.16-5.14 (m, 1H), 5.10-5.07 (m, 1H), 4.89-4.87 (m, 1H), 4.80-4.78 (m, 1H), 4.38-4.05 (m, 4H); $^{13}$C NMR (100 MHz, $CDCl_3$): $\delta$ 170.8, 170.7, 170.2, 169.8, 147.1, 129.7, 126.4, 96.5, 84.4, 74.4, 70.6, 68.9, 68.1, 67.6, 64.6, 62.6, 61.9, 53.4, 21.0, 20.8, 20.6; HRMS calcd for $C_{10}H_{13}N_3NaO_5$ $[M + Na]^+$ 278.0753, Found: 278.0754.

**Compound 6:** The glycal **1b** (100 mg, 0.37 mmol) was treated with $TMSN_3$ (0.10 mL, 0.74 mmol) by using the general procedure to afford **6** (61 mg, 59%) as a colorless oil: $R_f$ = 0.5 (hexane/EtOAc = 4:1); IR (neat) $\nu_{max}$: 2920, 2851, 2117, 1747, 1660, 1434, 1371, 1223 $cm^{-1}$; $^1$H NMR (500 MHz, $CDCl_3$): $\delta$ 6.45 (d, *J* = 6.3 Hz, 1H), 6.33 (d, *J* = 4.0 Hz, 1H), 5.55-5.23 (m, 3H), 4.94-4.91 (m, 1H), 4.72 (m, 1H), 4.35-4.10 (m, 8H), 3.83-3.79 (m, 1H), 2.16-2.00 (m, 3H, 4 isomers); $^{13}$C NMR (100 MHz, $CDCl_3$): $\delta$ 170.6, 170.4, 170.3, 170.2, 169.8, 169.5, 145.5, 98.9, 96.9, 87.2, 72.8, 69.5, 69.0, 68.6, 66.6, 66.2, 65.6, 63.9, 63.8, 62.2, 62.0, 60.9, 55.9, 53.1, 20.9, 20.8, 20.7, 20.6; HRMS calcd for $C_{10}H_{13}N_3NaO_5$ $[M + Na]^+$ 278.0753, Found: 278.0751.

**((2*R*,3*S*)-3-Acetoxy-3,6-dihydro-2*H*-pyran-2-yl)methyl acetate 7**: The glucal **1a** (100 mg, 0.37 mmol) was treated with $Et_3SiH$ (0.12 mL, 0.74 mmol) by using the general procedure to afford **7** (38 mg, 43%) as a colorless oil: $R_f$ = 0.4 (hexane/EtOAc = 4:1); $[\alpha]_D^{28}$ = +81.4 (*c* 0.70, $CHCl_3$); IR (neat) $\nu_{max}$: 2922, 2850, 1735, 1445, 1368, 1226, 1022 $cm^{-1}$; $^1$H NMR (500 MHz, $CDCl_3$): $\delta$ 5.93 (dd, *J* = 1.7, 10.3 Hz, 1H), 5.74 (dd, *J* = 2.3, 10.3 Hz, 1H), 5.24 (m, 1H), 4.21-4.14 (m, 4H), 3.73-3.69 (m, 1H), 2.09 (s, 3H), 2.06 (s, 3H); $^{13}$C NMR (125 MHz,

CDCl3): $\delta$ 170.7, 170.1, 126.5, 126.4, 115.3, 72.0, 62.7, 62.2, 20.8; HRMS calcd for $C_{15}H_{18}NaO_2$ [M + Na]$^+$ 237.0739, Found: 237.0732.

**(2*R*,3*S*,6*R*)-6-Allyl-2-(benzyloxymethyl)-3,6-dihydro-2*H*-pyran-3-ol (8):** The diacetate **2a** (500 mg, 2.0 mmol) was dissolved in dry methanol (10 mL), cooled to 0 °C, and then treated with anhydrous $K_2CO_3$ (1.38 g, 10.0 mmol). The reaction mixure was stirred at room temperature for 2 hours. The reaction mixture was passed through a silica plug and concentrated. The residue was dissolved in dry toluene (5 mL) and treated with *n*-$Bu_2SnO$ (747 mg, 3.0 mmol) at 140 °C for 6h by using a Dean–Stark apparatus. The reaction mixture was then cooled to room temperature, and $Et_3N$ (0.21 mL, 1.50 mmol), TBAI (812 mg, 2.2 mmol) and BnBr (0.95 mL, 8.0 mmol) were added. The resulting solution was heated to 120 °C for 2 h, poured into 1 N HCl (10 mL), and extracted with ethyl acetate (3 × 10 mL). The extracted material was washed with brine (1 × 20 mL), dried over $Na_2SO_4$, and concentrated under vacuum. The crude residue was purified by column chromatography to afford product **8** (355 mg, 69%) as a pale yellow liquid: $R_f$ = 0.6 (hexane/EtOAc = 4:1); $[\alpha]_D^{28}$ = -21.1 (*c* 1.80, $CH_2Cl_2$); IR (neat) $\nu_{max}$: 3430, 3032, 2924, 2870, 1641, 1496, 1453, 1255, 1091 cm$^{-1}$; $^1$H NMR (500 MHz, CDCl3): $\delta$ 7.36–7.27 (m, 5H), 5.87–5.77 (m, 3H), 5.12–5.06 (m, 2H), 4.58 (s, 2H), 4.22–4.18 (m, 1H), 4.10–4.07 (m, 1H), 3.72 (dd, *J* = 5.2, 8.5 Hz, 1H), 3.70 (td, *J* = 5.5, 6.7 Hz, 1H), 2.52 (*br* s, 1H), 2.47–2.41 (m, 1H), 2.32–2.26 (m, 1H); $^{13}$C NMR (125 MHz, CDCl3): $\delta$ 137.7, 134.5, 134.4, 130.4, 128.5, 128.0, 127.9, 127.8, 117.5, 73.7, 72.0, 71.7, 71.2, 65.8, 38.1; HRMS calcd for $C_{16}H_{20}NaO_3$ [M + Na]$^+$ 283.1310, Found: 283.1313.

**1-((2*R*,5*S*,6*R*)-6-(benzyloxymethyl)-5-hydroxy-5,6-dihydro-2*H*-pyran-2-yl)propan-2-one (9):** To a stirred solution of $PdCl_2$ (8 mg, 0.046 mmol) in DMF/$H_2O$ (4:1, 2 mL) was added CuCl (91 mg, 0.92 mmol) and stirred under $O_2$ (balloon) for 1 h. Then a solution of olefin **8** (120 mg, 0.46 mmol), dissolved in DMF (1 mL), was added, and again stirred for 6 h under

$O_2$. On completion of the reaction (TLC monitoring), the reaction mixture was poured into 1 N HCl (5 mL), followed by an extraction with ethyl acetate (3 × 5 mL). The combined organic extracts were washed with ice-water (1 × 10 mL), brine (1 × 10 mL) and then dried over $Na_2SO_4$. The solvent was removed under vacuum and crude residue was purified by column chromatography, to yield 98 mg (77%) of ketone **9** as a colorless oil: $R_f$ = 0.3 (hexane/EtOAc = 3:1); $[\alpha]_D^{28}$ = -1.4 (*c* 1.40, $CH_2Cl_2$); IR (neat) $\nu_{max}$: 3421, 2869, 1712, 1496, 1453, 1362, 1261, 1165, 1095, 1074 $cm^{-1}$; $^1H$ NMR (500 MHz, $CDCl_3$): $\delta$ 7.35–7.25 (m, 5H), 5.83 (ddd, *J* = 2.4, 4.6, 10.4 Hz, 1H), 5.78–5.74 (m, 1H), 4.66–4.62 (m, 1H), 4.56 (s, 2H), 4.08 (d, *J* = 5.8 Hz, 1H), 3.70–3.57 (m, 3H), 2.83 (dd, *J* = 8.2, 16.2 Hz, 1H), 2.69 (*br* s, 1H), 2.57 (dd, *J* = 5.5, 16.2 Hz, 1H), 2.17 (s, 3H); $^{13}C$ NMR (125 MHz, $CDCl_3$): $\delta$ 206.4, 137.7, 129.8, 128.5, 127.9, 127.8, 73.8, 72.1, 71.0, 68.8, 65.4, 46.9, 30.8; HRMS calcd for $C_{16}H_{20}NaO_4$ $[M + Na]^+$ 299.1259, Found: 299.1259.

***N*-((2*R*,3*R*,6*S*)-6-(Benzyloxymethyl)-2-(2-oxopropyl)-3,6-dihydro-2*H*-pyran-3-yl)-2,2,2-trichloroacetamide (10):** To a stirred solution of allylic alcohol **9** (110 mg, 0.40 mmol) in dry dichloromethane (3 mL) at 0 °C, was added trichloroacetonitrile (0.04 mL, 0.40 mmol), followed by DBU (0.06 mL, 0.40 mmol), and the mixture was stirred for 30 minutes at the same temperature. The solvent was removed by evaporation and the residue was passed through a silica plug followed by concentration of the filtrate. The crude trichloroacetimidate ($R_f$ 0.6 in hexane/EtOAc = 4:1) was then dissolved in dry xylene (3 mL) and $K_2CO_3$ (5 mg, 0.04 mmol) was added to it. The resulting mixture was heated under reflux overnight. The solvent was then evaporated and the residue purified by column chromatography to afford 120 mg (72% over two steps) of the rearranged product **10** as a pale yellow liquid: $R_f$ = 0.7 (hexane/EtOAc = 4:1); $[\alpha]_D^{28}$ = -84.0 (*c* 0.25, $CH_2Cl_2$); IR (neat) $\nu_{max}$: 2922, 1710, 1499, 1062, 820 $cm^{-1}$; $^1H$ NMR (500 MHz, $CDCl_3$): $\delta$ 7.36–7.28 (m, 5H), 6.66 (d, *J* = 8.8 Hz, 1H),

6.02 (s, 2H), 4.60–4.51 (m, 3H), 4.43 (dd, *J* = 4.6, 6.1 Hz, 1H), 4.32–4.28 (m, 1H), 3.68 (dd, *J* = 7.0, 10.1 Hz, 1H), 3.56 (dd, *J* = 4.3, 10.1 Hz, 1H), 2.66 (dd, *J* = 7.6, 17.1 Hz, 1H), 2.58 (dd, *J* = 4.9, 17.1 Hz, 1H), 2.16 (s, 3H); $^{13}$C NMR (125 MHz, $CDCl_3$): $\delta$ 205.3, 162.0, 137.9, 131.5, 128.5, 127.8, 124.9, 73.6, 69.9, 67.9, 46.3, 44.7, 30.7; HRMS calcd for $C_{18}H_{20}Cl_3NNaO_4$ $[M + Na]^+$ 442.0356, Found: 442.0352.

**Benzyl (2R,3R,6S)-6-(benzyloxymethyl)-2-(2-oxopropyl)-3,6-dihydro-2*H*-pyran-3-ylcarbamate (11):** The trichloroacetamide **10** (110 mg, 0.26 mmol) was dissolved in THF (1 mL), 6 N HCl (2 mL) was added, and the resulting solution heated under reflux overnight. Then the solution was cooled to 0 °C, quenched by the careful addition of saturated $NaHCO_3$ (5 mL), and extracted with EtOAc (3 × 5 mL). The extracts were dried over $Na_2SO_4$, and concentrated under vacuum. ($R_f$ = 0.2 (hexane/EtOAc = 1:1)). The crude product was then dissolved in EtOAc (3 mL), benzyl chloroformate (0.14 mL, 0.52 mmol, 50% in toluene) was added followed by $Na_2CO_3$ (83 mg, 0.78 mmol), and the mixture stirred for 2 h at room temperature. The reaction mixture was diluted with 5 mL water and extracted with EtOAc (3 × 5 mL). Extracts were washed with brine (1 × 10 mL), dried over $Na_2SO_4$, and concentrated under vacuum. The crude residue was purified by column chromatography to yield 77 mg of **11** (74% over 2 steps) as a colorless oil: $R_f$ = 0.4 (hexane/EtOAc = 4:1); $[\alpha]_D^{28}$ = -90.0 (*c* 0.50, $CH_2Cl_2$); IR (neat) $\nu_{max}$: 3323, 2922, 1715, 1498, 1454, 1363, 1233, 1109, 1049 cm$^{-1}$; $^1$H NMR (500 MHz, $CDCl_3$): $\delta$ 7.36–7.27 (m, 10H, aromatic), 5.99 (ddd, *J* = 2.1, 6.1, 10.1 Hz, 1H, H-3), 5.87 (dd, *J* = 3.1, 10.1 Hz, 1H, H-4), 5.12–5.07 (m, 2H, $NHCO_2CH_2Ph$), 4.85 (d, *J* = 9.8 Hz, 1H, -N*H*CO), 4.58 (d, *J* = 12.2 Hz, 1H, $OCH_2Ph$), 4.52 (d, *J* = 12.2 Hz, 1H, $OCH_2Ph$), 4.41–4.38 (m, 1H, H-1), 4.35–4.32 (m, 1H, H-5), 4.04–4.00 (m, 1H, H-2), 3.64 (dd, *J* = 7.0, 10.4 Hz, 1H, H-6), 3.50 (dd, *J* = 4.6, 10.4 Hz, IH, H-6'), 2.62 (dd, *J* = 8.2, 16.8 Hz, 1H, H-1'), 2.55 (dd, *J* = 4.6, 16.8 Hz, 1H, H-1''), 2.11 (s, 3H, $CH_3CO$-); $^{13}$C NMR (125

MHz, $CDCl_3$): $\delta$ 206.1, 156.2, 138.0, 136.4, 129.7, 128.6, 128.4, 128.3, 128.1, 127.8, 127.7, 126.4, 73.5, 73.3, 70.0, 68.1, 67.0, 46.1, 45.1, 30.7; HRMS calcd for $C_{24}H_{28}NO_5$ $[M + H]^+$ 410.1967, Found: 410.1967.

**Benzyl (3a*R*,4*R*,6*R*,7*S*,7a*R*)-4-(benzyloxymethyl)-2,2-dimethyl-6-(2-oxopropyl)tetrahyd-ro-3a*H*-[1,3]dioxolo[4,5-*c*]pyran-7-ylcarbamate (12):**The olefin **11** (50 mg, 0.12 mmol) was dissolved in an acetone/$H_2O$/*t*-BuOH solvent system (3:1:1, 3 mL), *N*-methylmorpholine *N*-oxide (17 mg, 0.14 mmol) and a catalytic amount of $OsO_4$ (0.1 mL, 1% w/w soln in *t*-BuOH) were added in succession, and the mixture was stirred at room temperature for 24 h. On completion (TLC monitoring) the reaction was treated with a saturated $Na_2S_2O_5$ solution (3 mL) and then extracted with ethyl acetate (3 × 3 mL). Organic extracts were washed with brine (1 × 5 mL), dried over $Na_2SO_4$, and concentrated. The crude residue was used for the next step without purification: $R_f$ = 0.3 (hexane/EtOAc = 1:1).

The crude diol was dissolved in dry $CH_2Cl_2$ (2 mL) and cooled to 0 °C. Then, 2,2-dimethoxypropane (0.02 mL, 0.14 mmol) was added, followed by PTSA (2 mg, 0.01 mmol), and stirred at room temperature for 1 h. The reaction mixture was diluted with $CH_2Cl_2$ (2 mL) and a saturated $NaHCO_3$ solution (3 mL) was added. Extraction was carried out with $CH_2Cl_2$ (3 × 3 mL), and extracts were dried over $Na_2SO_4$. The removal of the solvent under vacuum provided a crude residue, which was purified by column chromatography to furnish 48 mg (82%) of the compound **12** as a colorless oil: $R_f$ = 0.4 (hexane/EtOAc = 4:1); $[\alpha]_D^{28}$ = -6.7 (*c* 0.15, $CH_2Cl_2$); IR (neat) $\nu_{max}$: 3331, 2924, 2854, 1715, 1521, 1454, 1381, 1214, 1055 $cm^{-1}$; $^1H$ NMR (500 MHz, $CDCl_3$): $\delta$ 7.38–7.26 (m, 10H, aromatic), 5.09 (s, 2H, -$CO_2CH_2$Ph), 5.05 (d, $J$ = 7.3 Hz, 1H, -N*H*), 4.63 (m, 1H, H-1), 4.57 (d, $J$ = 12.2 Hz, 1H, -OC$H_2$Ph), 4.52 (d, $J$ = 12.2 Hz, 1H, -OC$H_2$Ph), 4.32 (dd, $J$ = 2.4, 7.3 Hz, 1H, H-4), 4.26–4.25 (m, 1H, H-5), 3.94-3.92 (m, 1H, H-3), 3.68–3.60 (m, 3H, H-2, H-6, H-6'), 2.64 (dd, $J$ = 8.7, 15.9 Hz, 1H, H-

1'), 2.45 (dd, $J$ = 5.5, 15.9 Hz, 1H, H-1"), 2.13 (s, 3H, C$H_3$CO-), 1.52 (s, 3H), 1.25 (s, 3H); $^{13}$C NMR (125 MHz, $CDCl_3$): $\delta$ 205.7, 155.9, 138.1, 136.1, 128.6, 128.5, 128.4, 128.3, 128.2, 127.7, 109.3, 73.5, 71.5, 70.4, 69.5, 67.2, 65.5, 52.7, 45.5, 30.3, 26.9, 24.3; HRMS calcd for $C_{27}H_{33}NNaO_7$ [M + Na]$^+$ 506.2155, Found: 506.2155.

## Copies of NMR spectra:

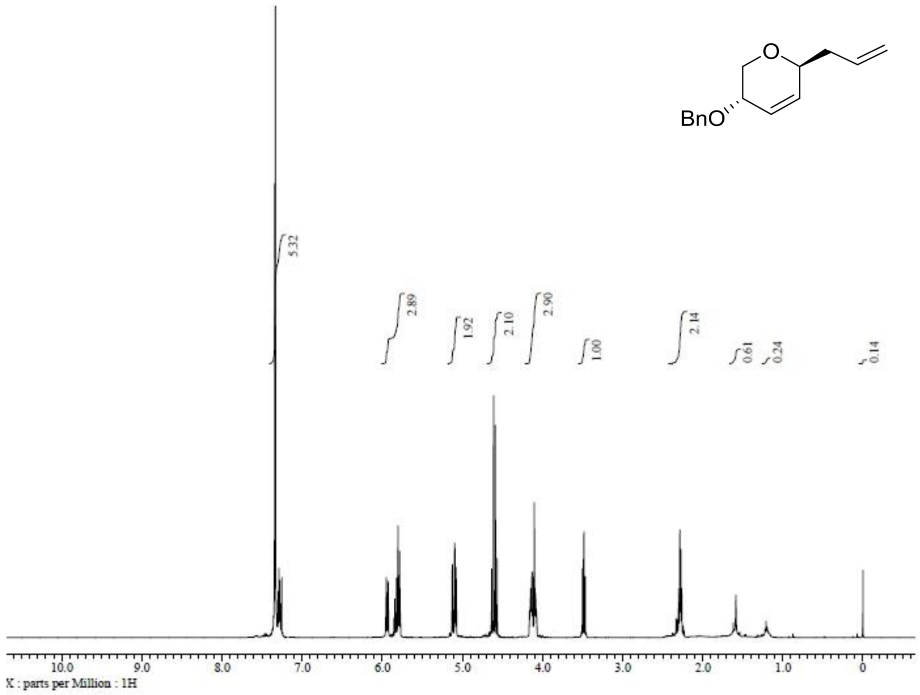


**Figure S1**: $^{1}$H NMR (500 MHz) spectrum of compound **2g**.

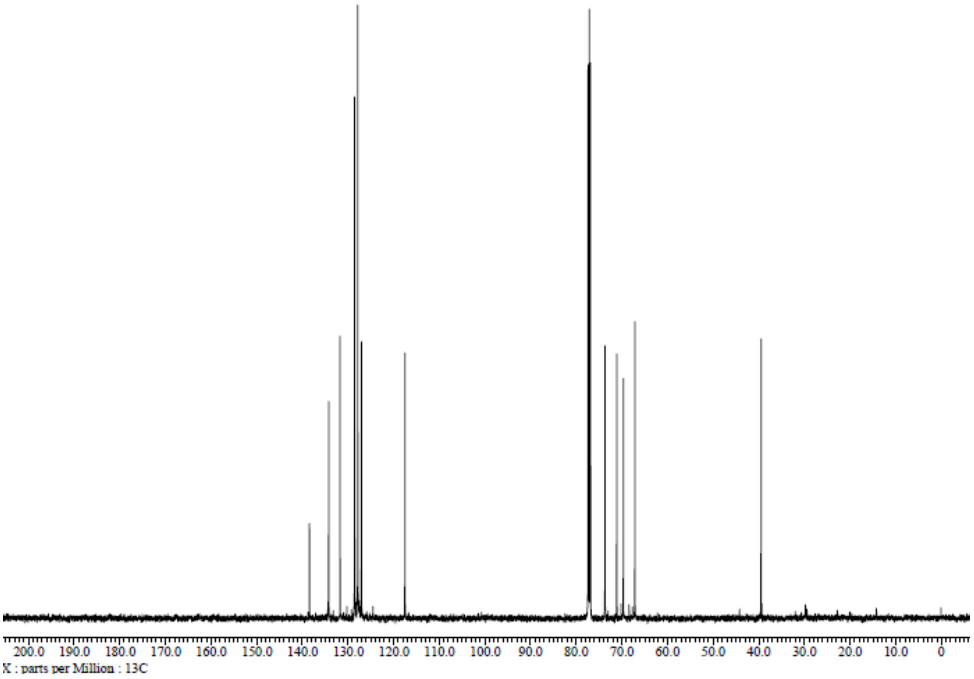


**Figure S2**: $^{13}$C NMR (125 MHz) spectrum of compound **2g**.

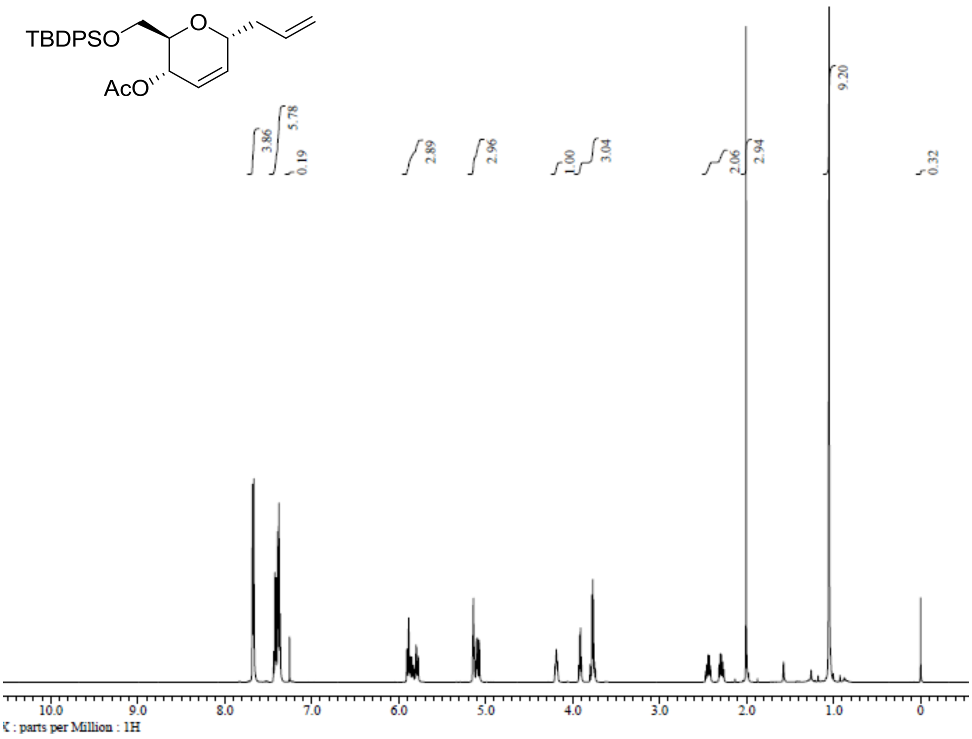


**Figure S3**: $^{1}$H NMR (500 MHz) spectrum of compound **2i**.

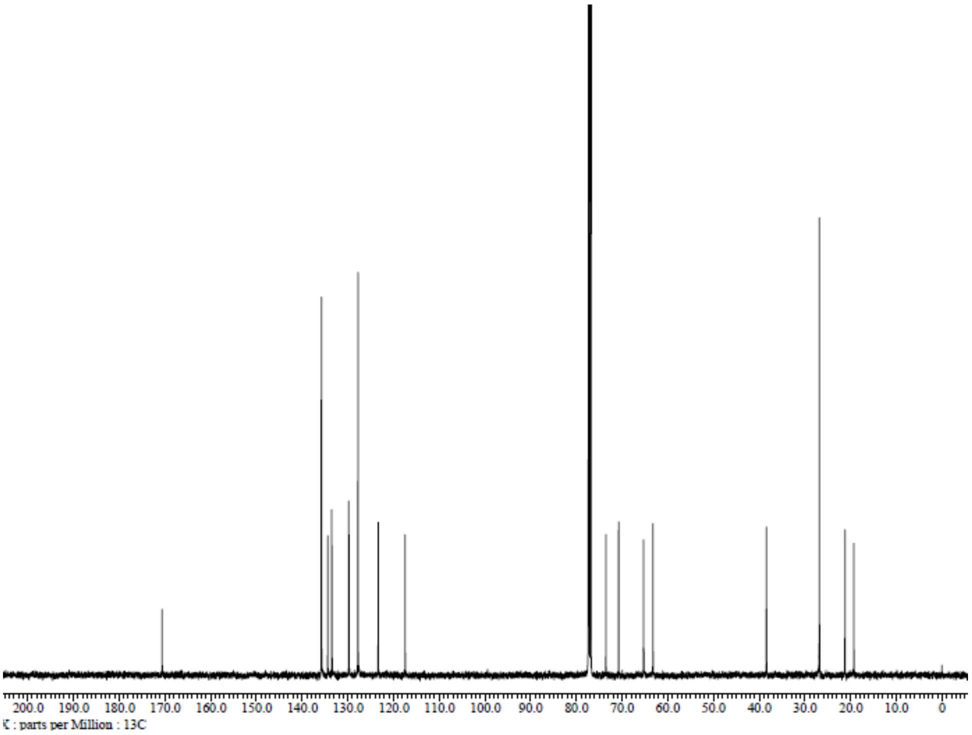


**Figure S4**: $^{13}$C NMR (125 MHz) spectrum of compound **2i**.

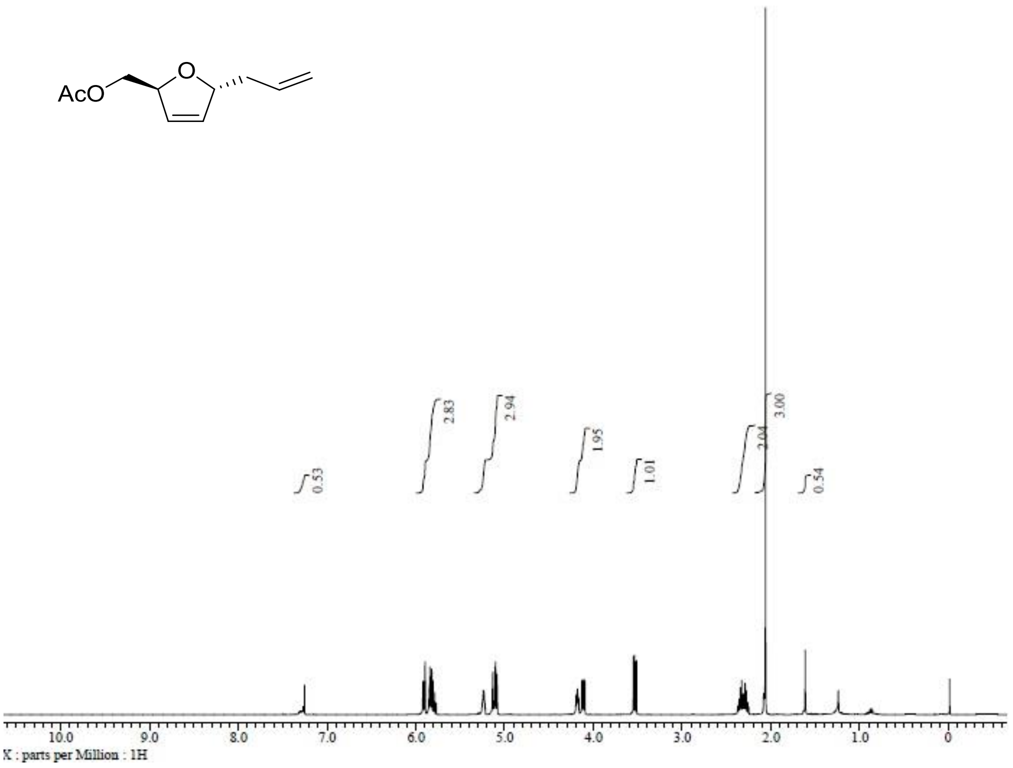


**Figure S5**: $^{1}$H NMR (500 MHz) spectrum of compound **2l**.

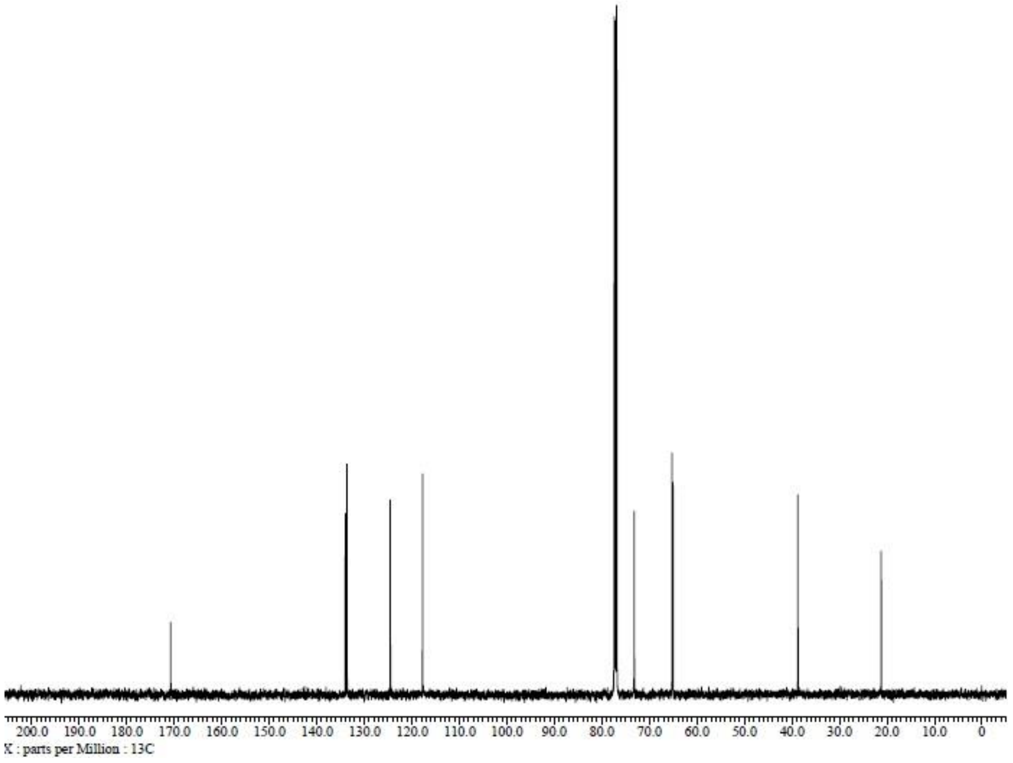


**Figure S6**: $^{13}$C NMR (125 MHz) spectrum of compound **2l**.

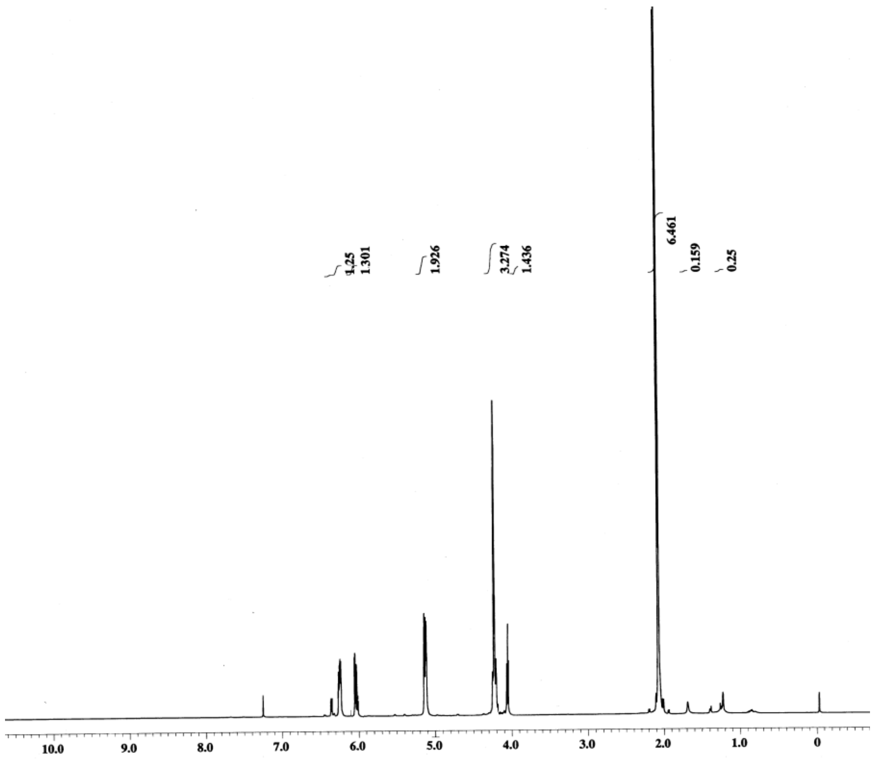


**Figure S7**: $^{1}$H NMR spectrum (500 MHz) of compound **3**.

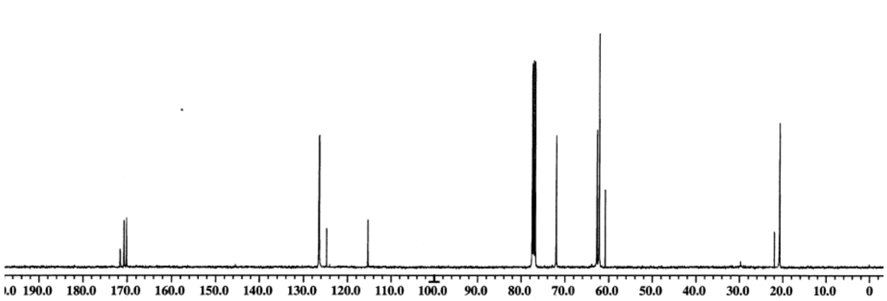


**Figure S8**: $^{13}$C NMR spectrum (100 MHz) of compound **3**.

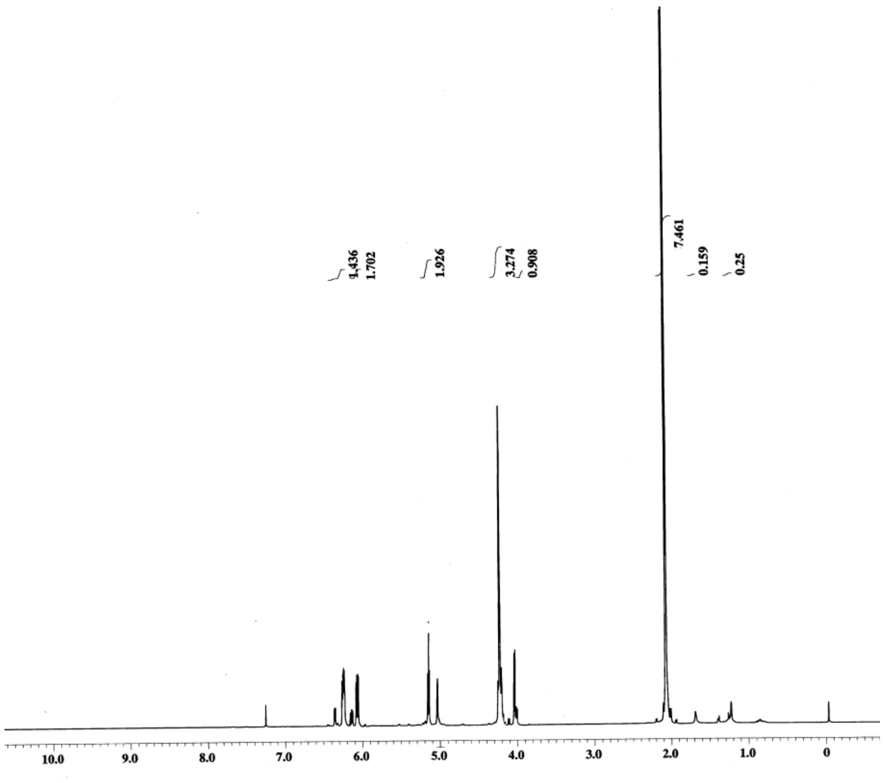


**Figure S9**: $^{1}$H NMR spectrum (500 MHz) of compound **4**.

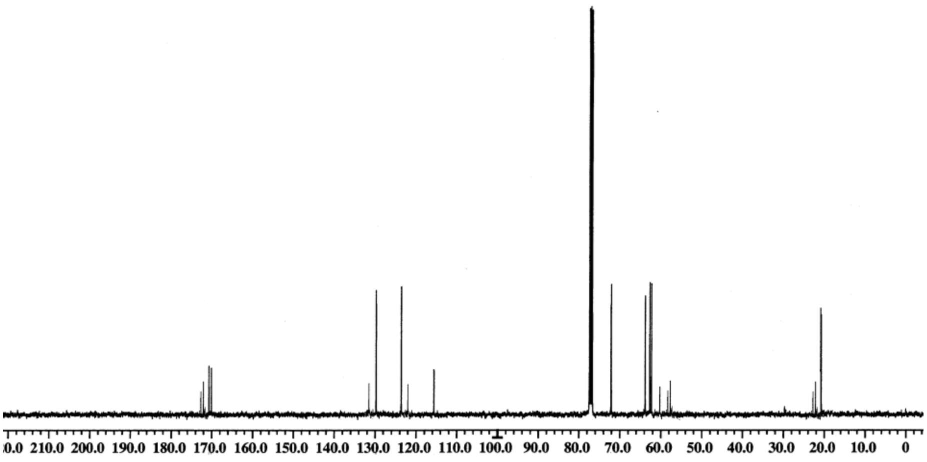


**Figure S10**: $^{13}$C NMR spectrum (100 MHz) of compound **4.**

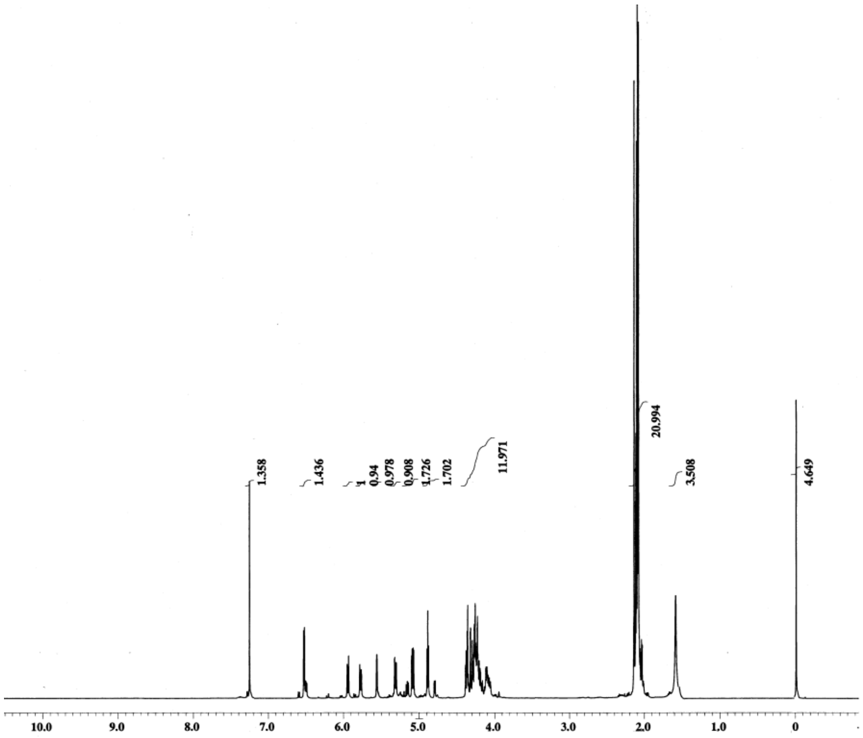

**Figure S11**: $^{1}$H NMR spectrum (500 MHz) of compound **5**.

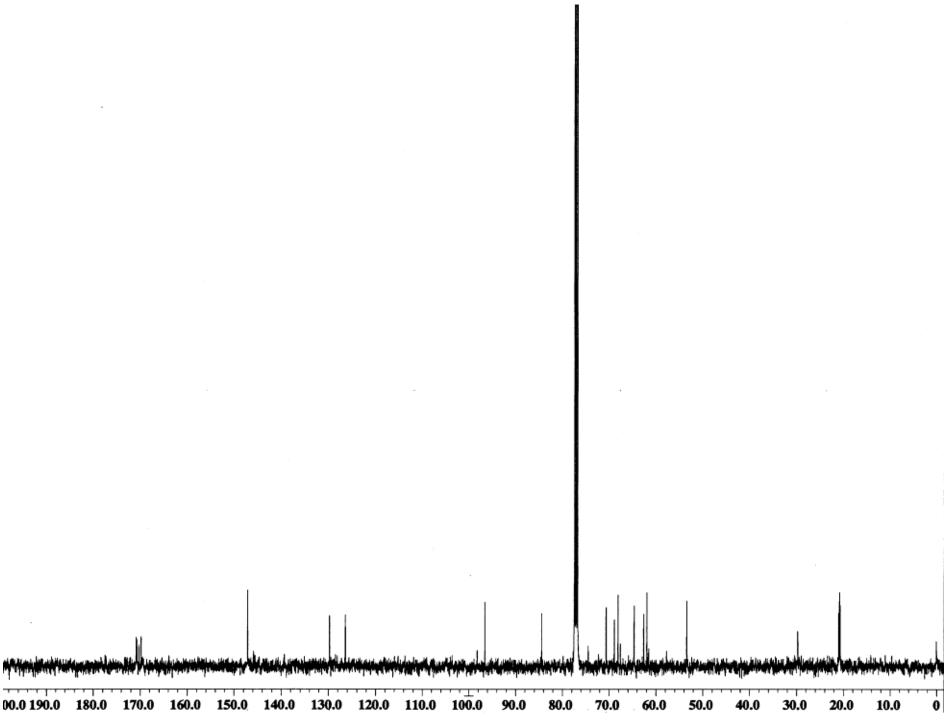

**Figure S12**: $^{13}$C NMR spectrum (100 MHz) of compound **5**.

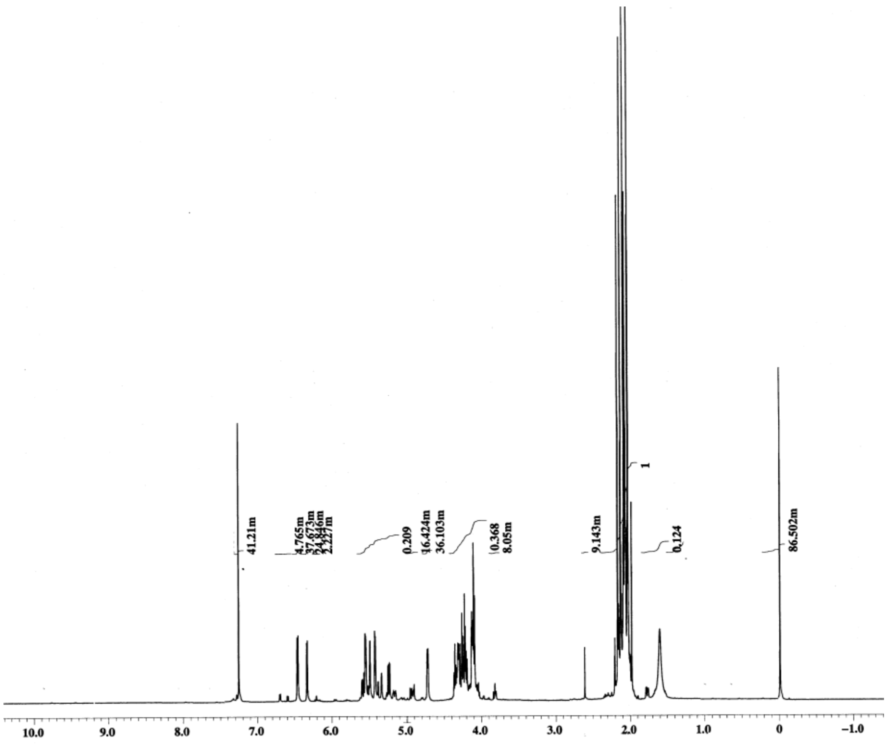

**Figure S13**: $^{1}H$ NMR spectrum (500 MHz) of compound **6**.

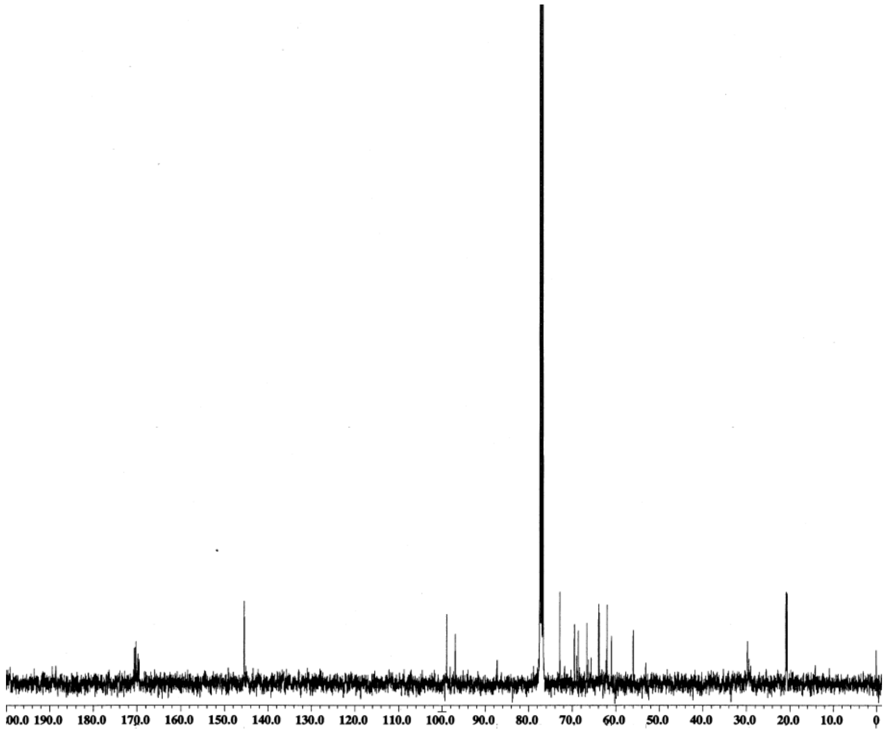

**Figure S14**: $^{13}C$ NMR spectrum (100 MHz) of compound **6**.

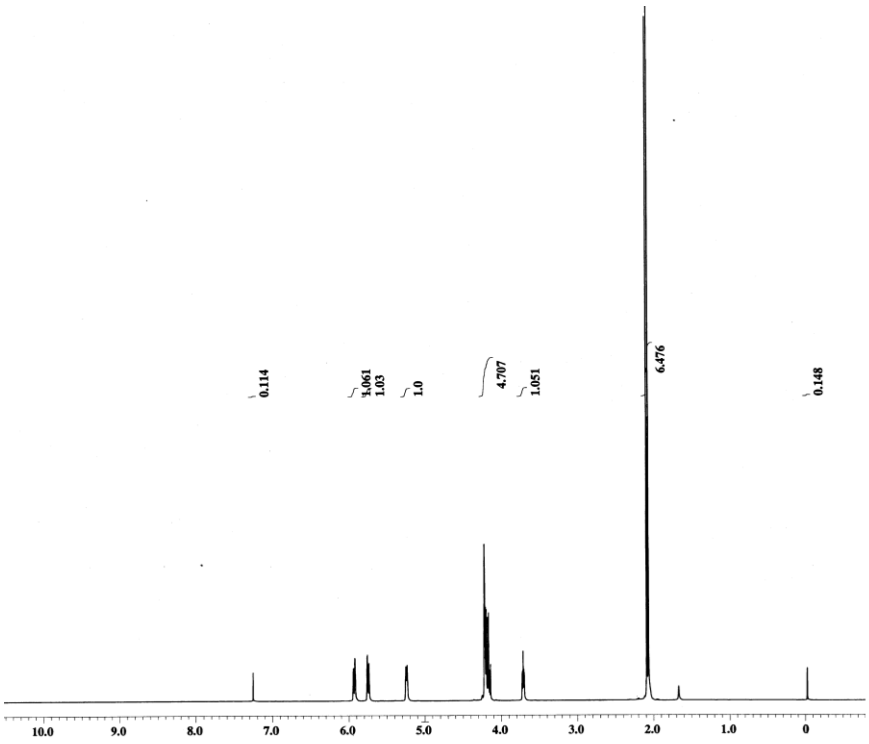


**Figure S15**: $^{1}$H NMR spectrum (500 MHz) of compound **7**.

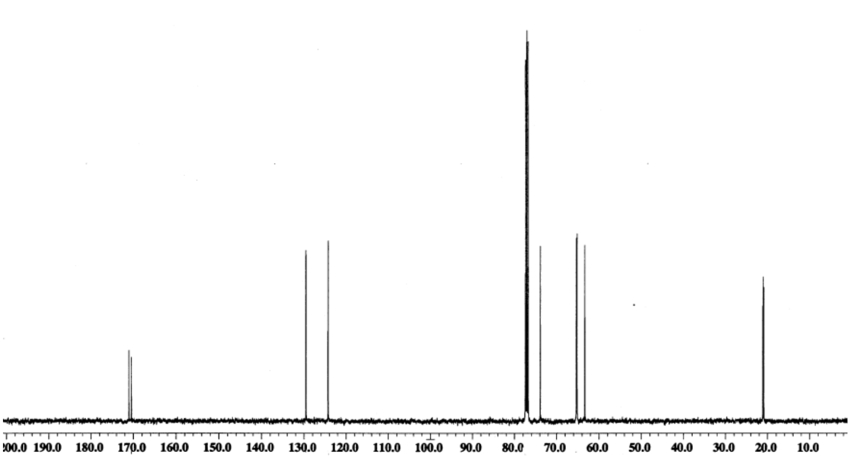


**Figure S16:** $^{13}$C NMR spectrum (100 MHz) of compound **7**.

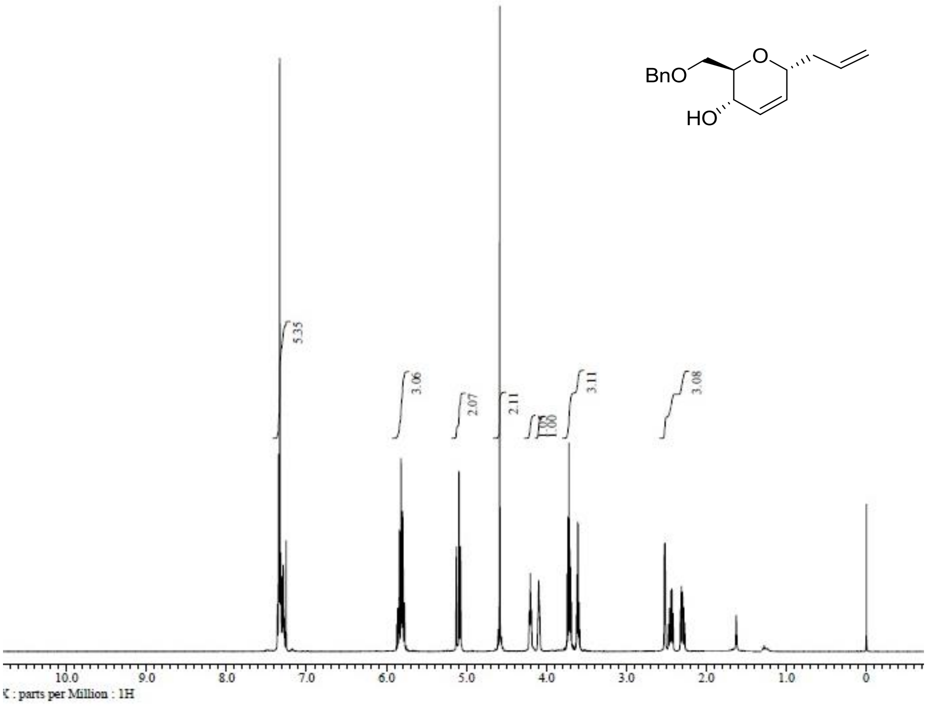


**Figure S17**: $^{1}$H NMR (500 MHz) spectrum of compound **8**.

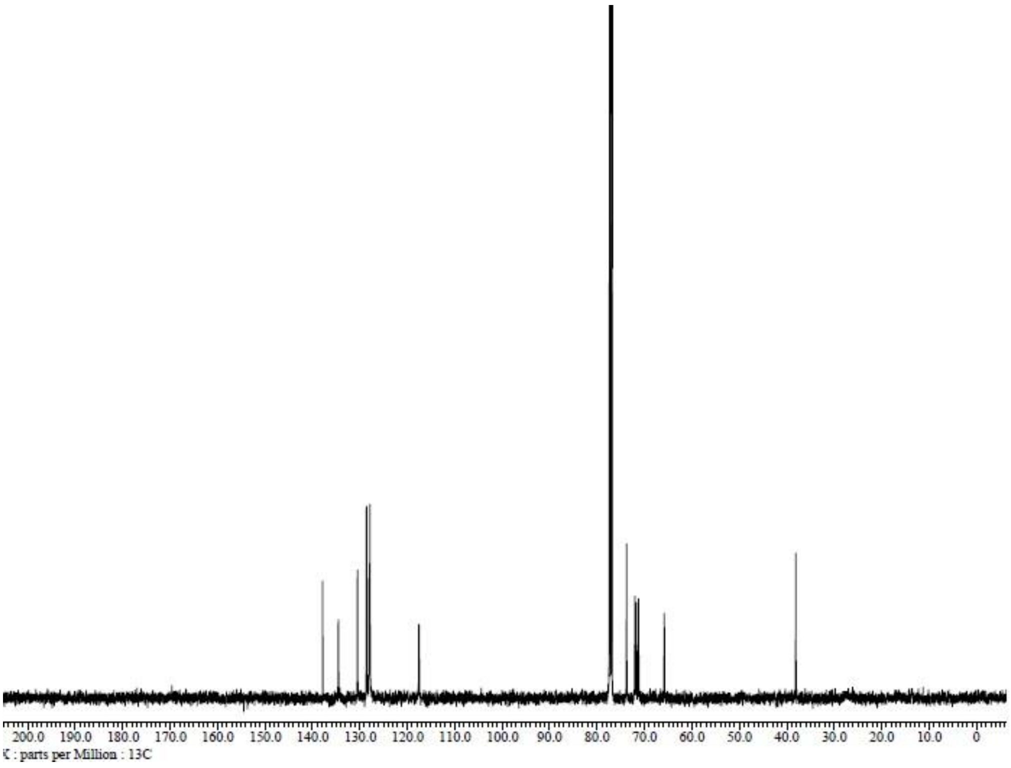


**Figure S18**: $^{13}$C NMR (500 MHz) spectrum of compound **8**.

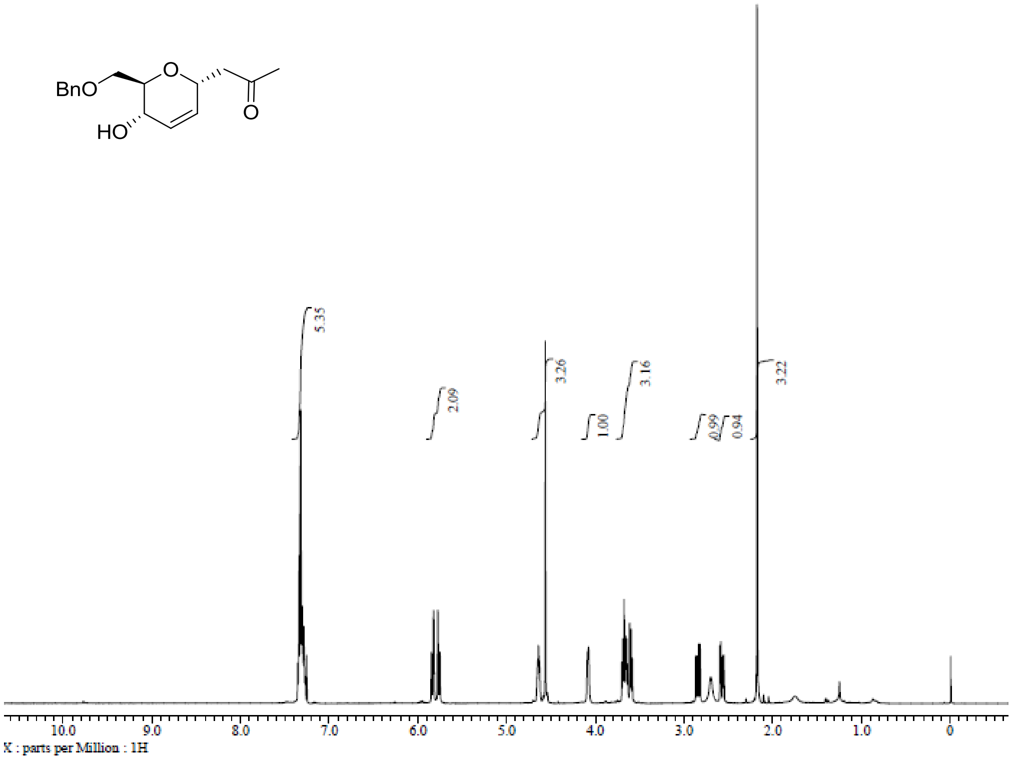


**Figure S19**: $^{1}$H NMR (500 MHz) spectrum of compound **9**.

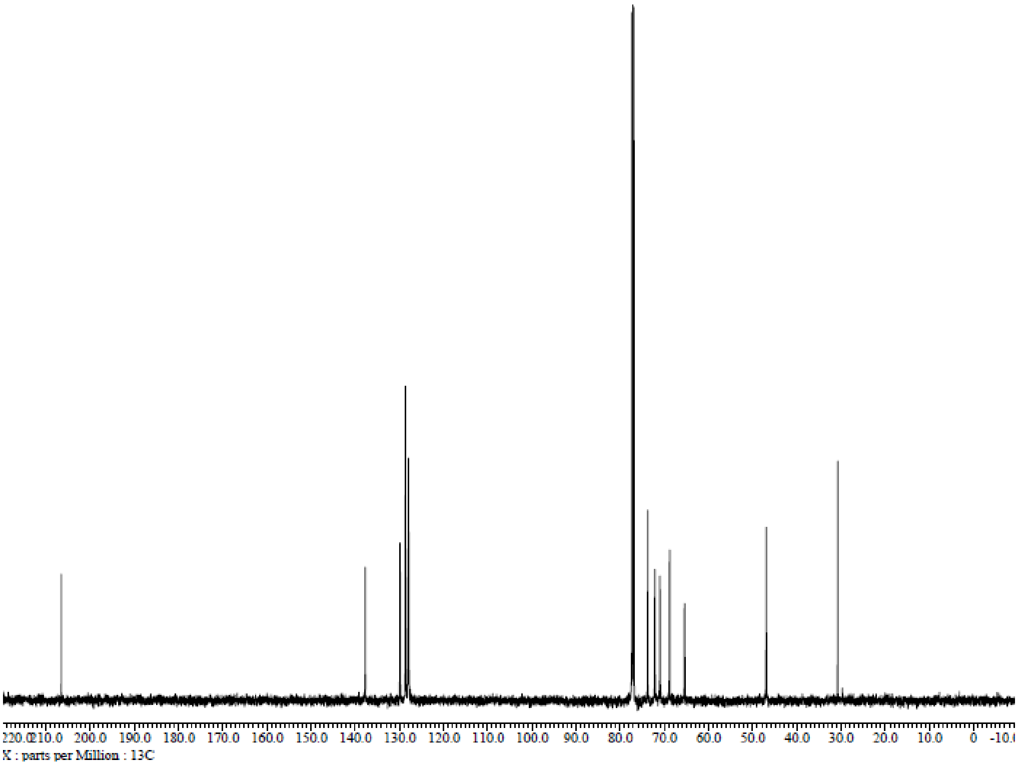


**Figure S20**: $^{13}$C NMR (125 MHz) spectrum of compound **9**.

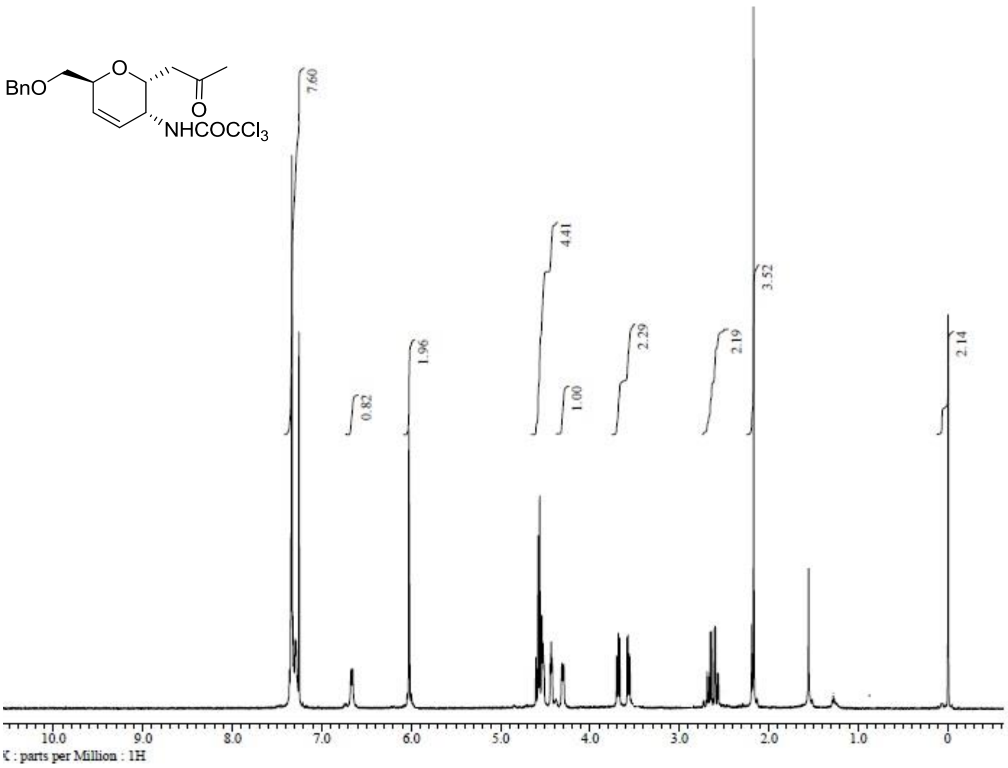


**Figure S21**: $^{1}$H NMR (500 MHz) spectrum of compound **10**.

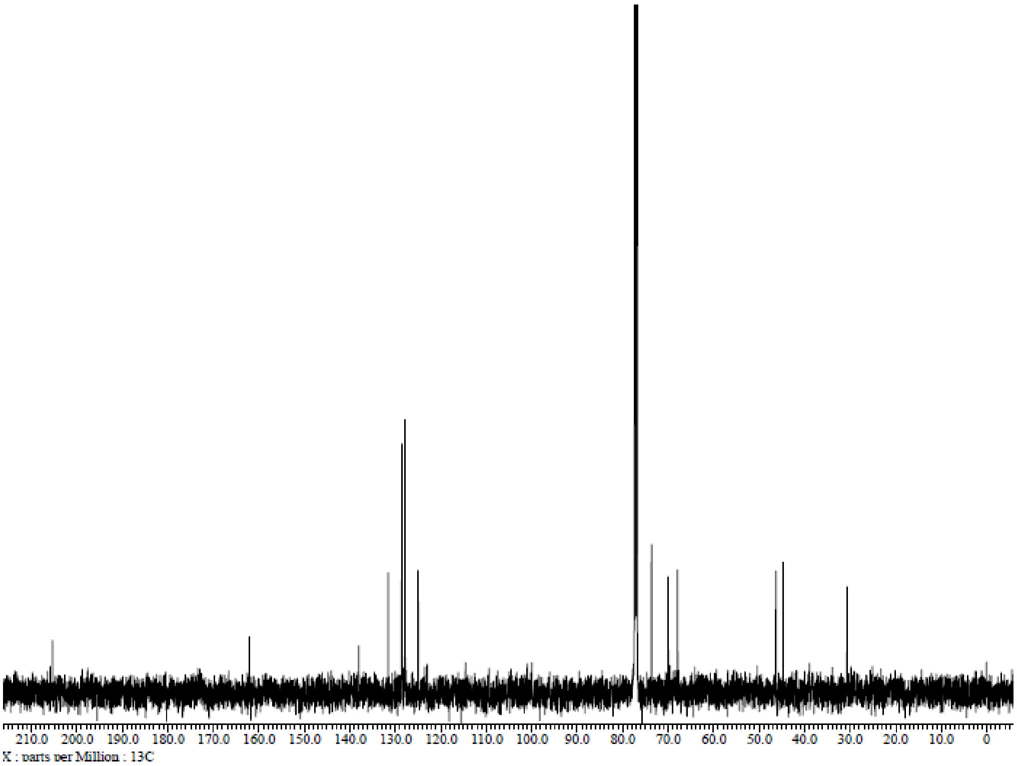


**Figure S22**: $^{13}$C NMR (125 MHz) spectrum of compound **10**.

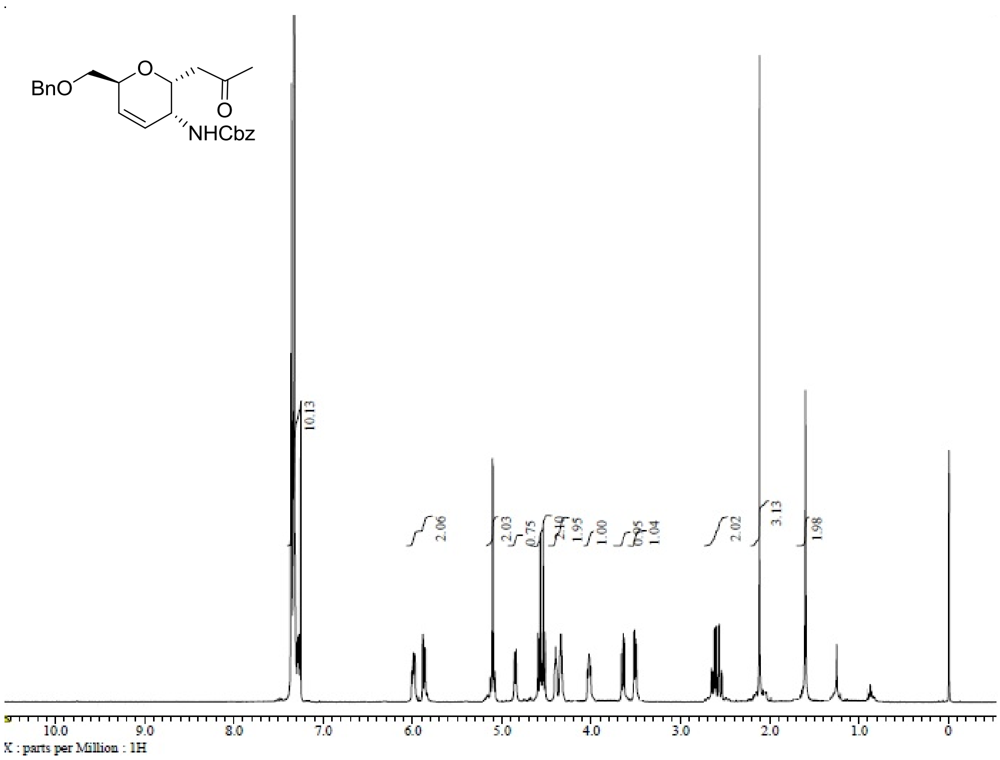


**Figure S23**: $^{1}$H NMR (500 MHz) spectrum of compound **11**.

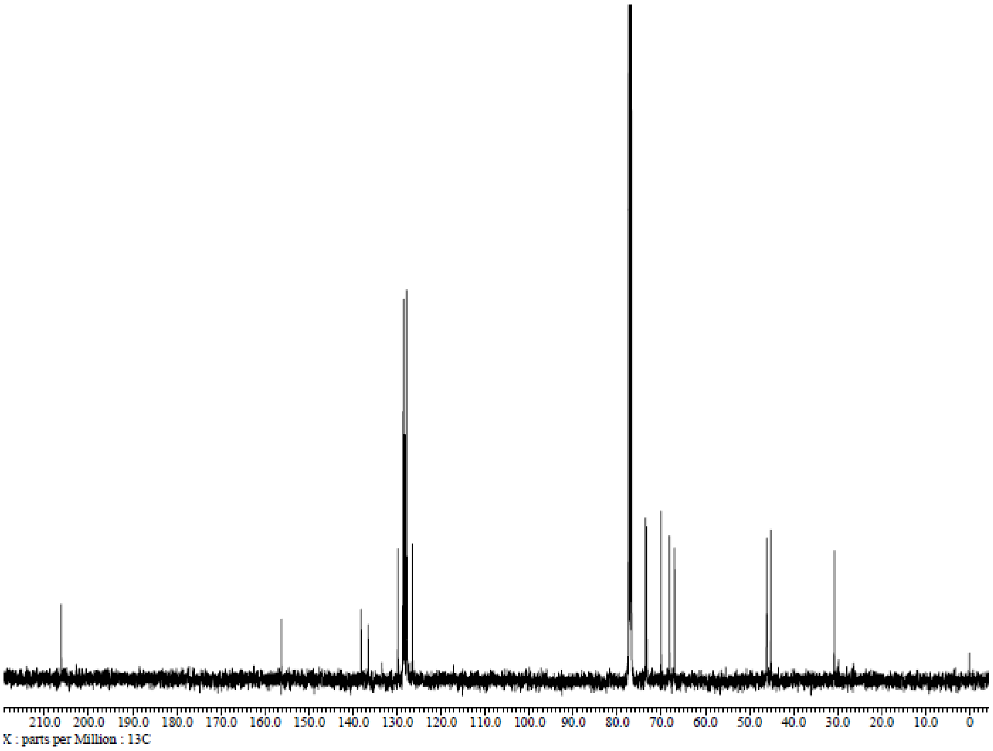


**Figure S24**: $^{13}$C NMR (125 MHz) spectrum of compound **11**.

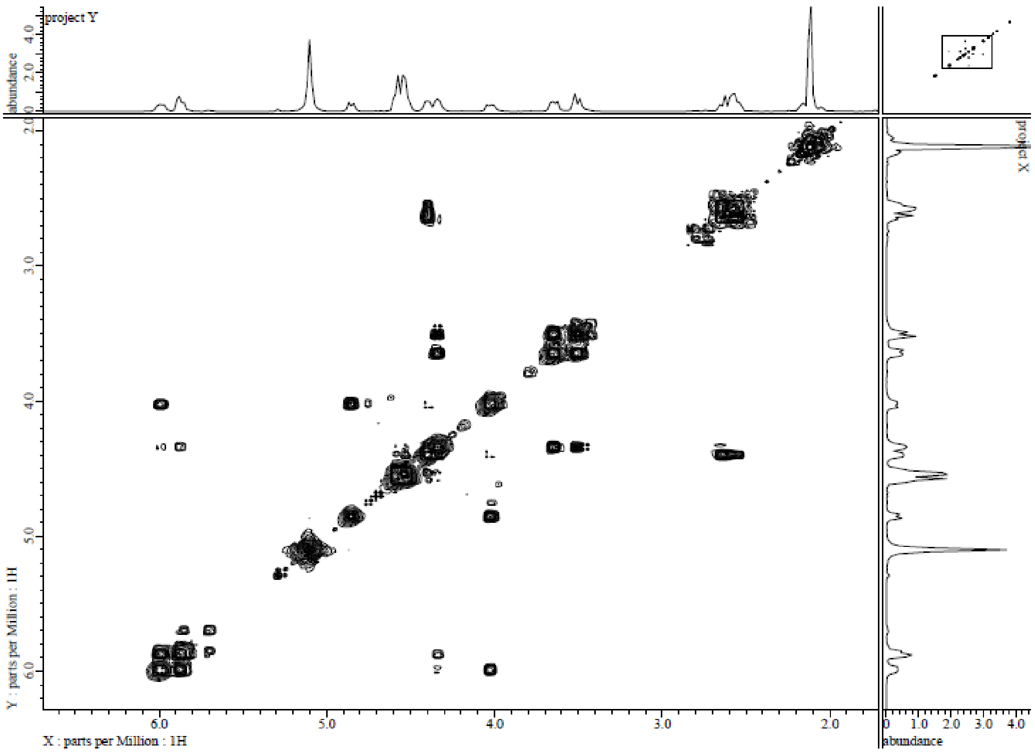


**Figure S25**: ¹H-¹H COSY spectrum of compound **11**.

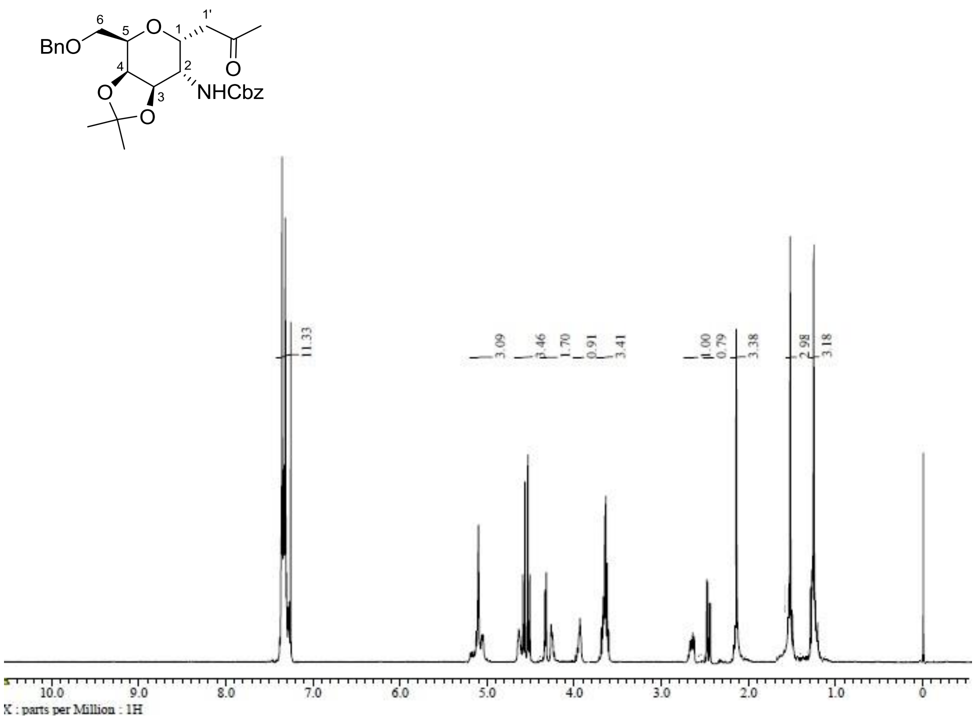


**Figure S26**: ¹H NMR (500 MHz) spectrum of compound **12**.

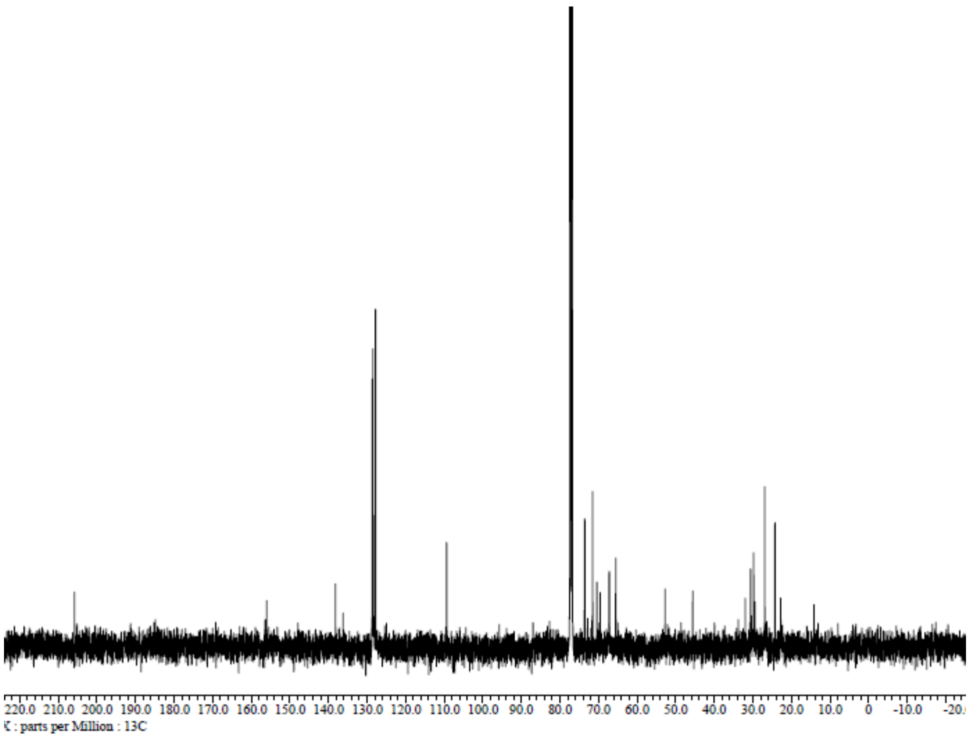


**Figure S27**: $^{13}$C NMR (125 MHz) spectrum of compound **12**.

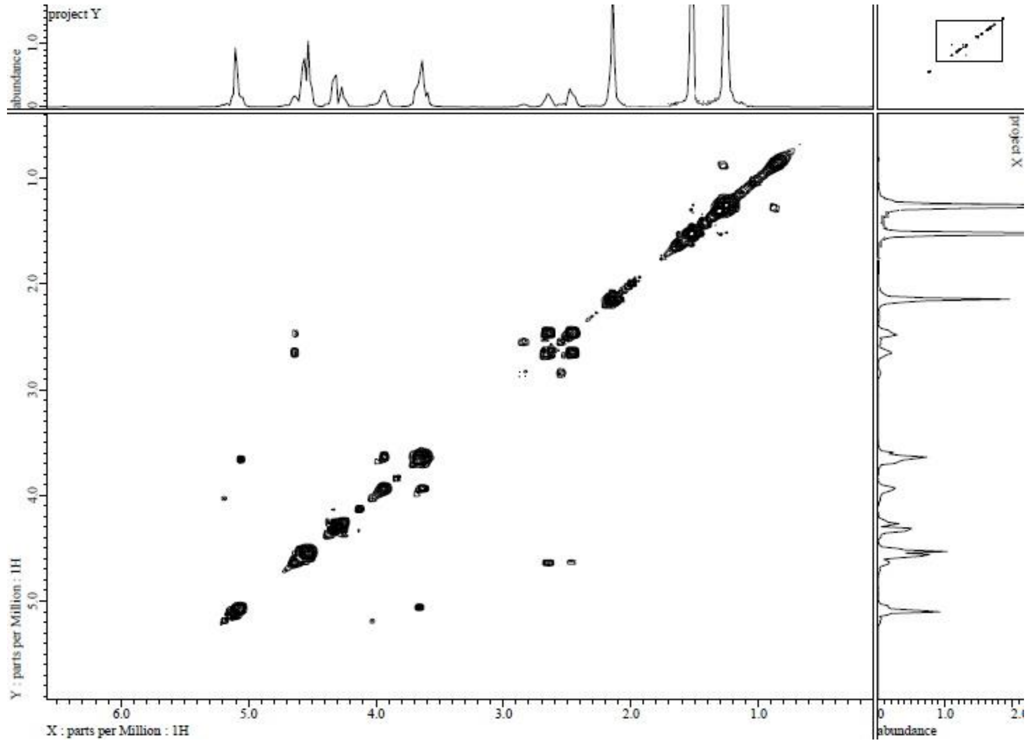


**Figure S28**: $^{1}$H-$^{1}$H COSY (500 MHz) spectrum of compound **12**.

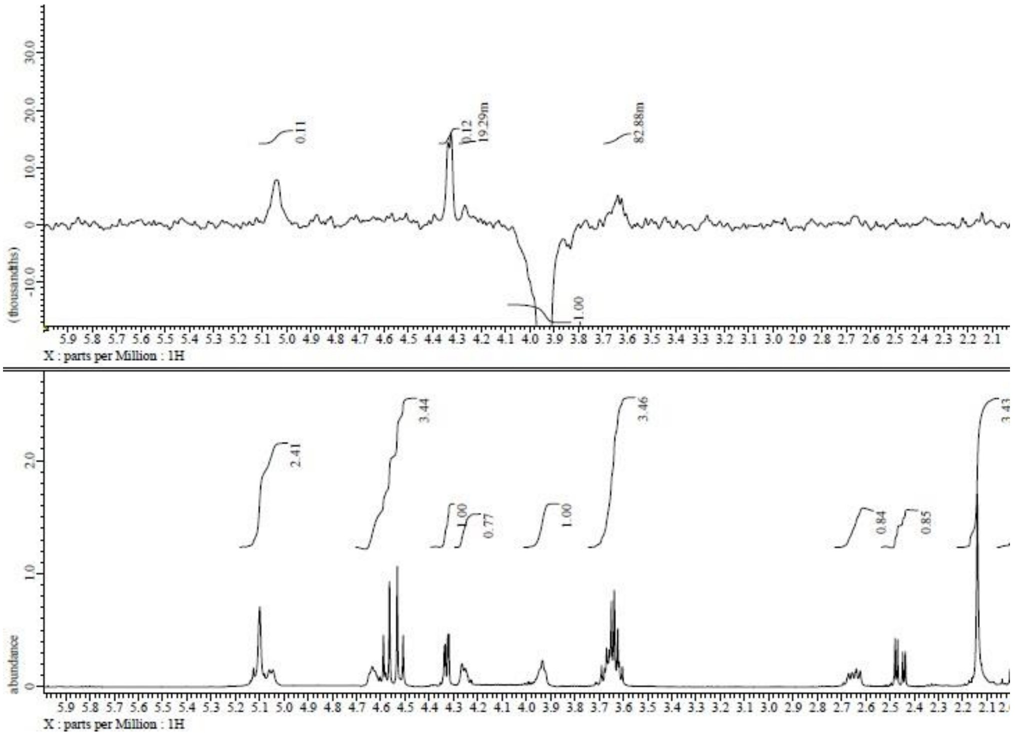


**Figure S29:** nOe spectrum (irradiation of H-4) of compound **12**.

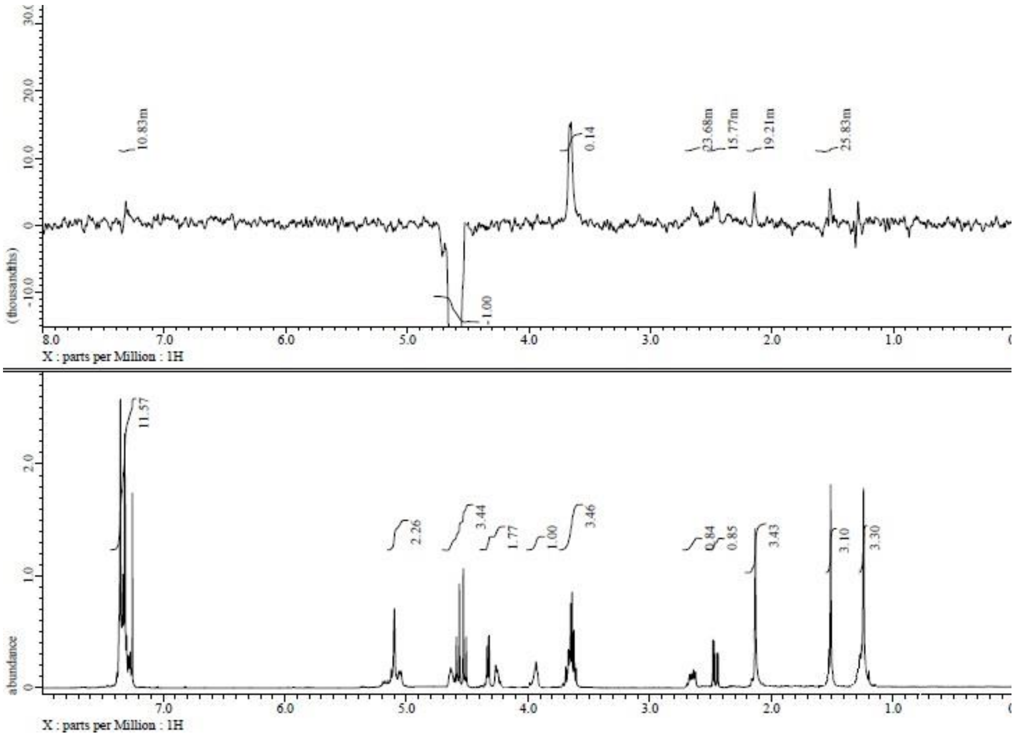


**Figure S30:** nOe spectrum (irradiation of H-2) of compound **12**.

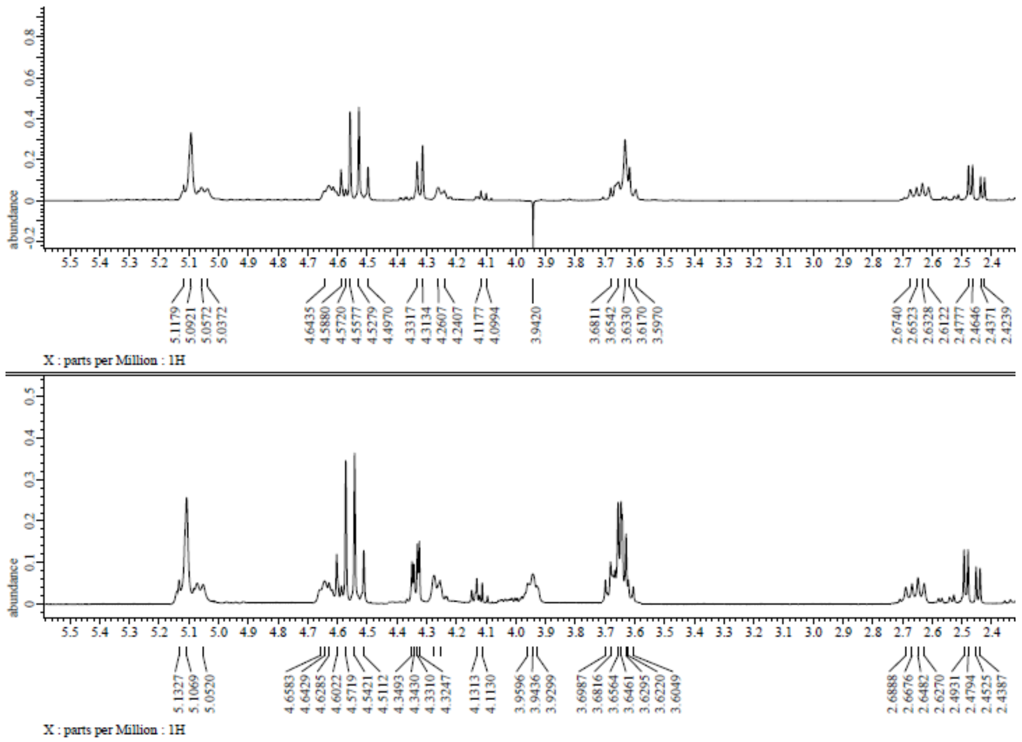


**Figure S31:** Homonuclear decoupling spectrum (400 MHz, H-3) of compound **12**.

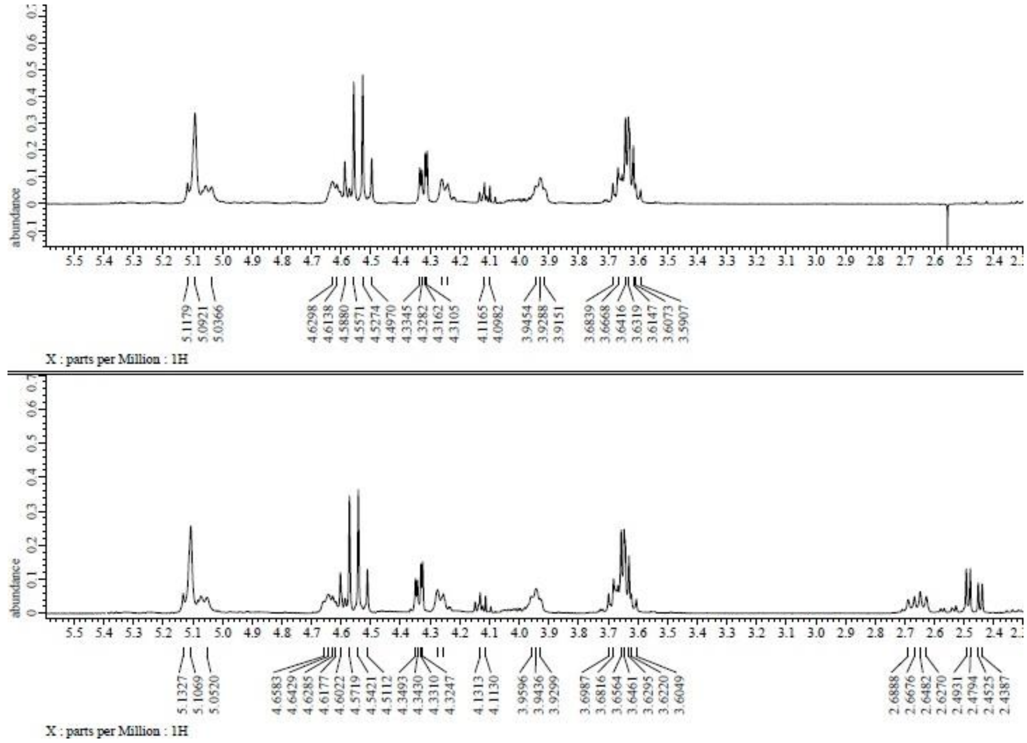


**Figure S32:** Homonuclear decoupling spectrum (400 MHz, H-1') of compound **12**.
